# Lung inflammation and lack of genotoxicity in the comet and micronucleus assays of industrial multiwalled carbon nanotubes Graphistrength^©^ C100 after a 90-day nose-only inhalation exposure of rats

**DOI:** 10.1186/s12989-015-0096-2

**Published:** 2015-07-10

**Authors:** Daniela Pothmann, Sophie Simar, Detlef Schuler, Eva Dony, Stéphane Gaering, Jean-Loïc Le Net, Yoshi Okazaki, Jean Michel Chabagno, Cécile Bessibes, Julien Beausoleil, Fabrice Nesslany, Jean-François Régnier

**Affiliations:** Harlan Laboratories Ltd, Zelgliweg 1, 4452 Itingen, Switzerland; Laboratoire de Toxicologie Génétique, Institut Pasteur de Lille, 1 rue du Pr. Calmette, 59019 Lille, Cedex France; Harlan Cytotest Cell Research GmbH, In den Leppsteinwiesen 19, 64380 Rossdorf, Germany; Le Net Pathology Consulting, 18 rue Henry Dunant, 37400 Amboise, France; AnaPath GmbH, Buchsweg 56, 4625 Oberbuchsiten, Switzerland; Arkema France, Groupement de Recherches de Lacq (GRL), Laboratoires d’analyse de surface et microscopie et de chimie analytique, 64170 Lacq, France; UDSL, EA 4483, Département Toxicologie-Santé Publique-Environnement, Faculté de Pharmacie, 1 rue du Pr. Laguesse, 59019 Lille, France; Arkema France, Département Toxicologie et Environnement, 420 rue d’ Estienne d’ Orves, 92705 Colombes, France

**Keywords:** Multiwalled carbon nanotubes, Comet assay, Micronucleus assay, Genotoxicity, Subchronic, Inhalation, Toxicity, NOAEC

## Abstract

**Background:**

Graphistrength^**©**^ C100 multiwalled carbon nanotubes (MWCNT) provide superior electrical and mechanical properties for various applications. The evaluation of the intrinsic hazard properties of Graphistrength^©^ C100 is an essential step for safe use. A general feature of multiwalled carbon nanotubes after inhalation or intratracheal exposures is the induction of an inflammatory reaction in the lungs sometimes associated with local genotoxic effects.

**Methods:**

After investigating different parameters for the aerosol generation and performing a 5-day inhalation range finding study, male and female Wistar rats were exposed nose-only for 90 days to target concentrations of 0.05, 0.25 and 5.0 mg/m^3^ air of Graphistrength^**©**^ C100 and sacrificed 24 h and 90 days after the last exposure. Broncho-alveolar lavage fluid (BALF) was also collected and analyzed for inflammatory parameters. Twenty-four hours post-exposure, chromosomal aberrations in the bone marrow cells were evaluated by the micronucleus test and DNA damages in the lung, kidney and liver cells by both the standard and the human 8-oxoguanine DNA N-glycosylase 1 (hOGG1)-modified comet assay. All studies were performed according to the OECD test guidelines.

**Results:**

An inflammatory lung reaction and the release of inflammatory factors in the BALF were observed in all rats exposed to 5.0 mg/m^3^, associated with changes in the differential white blood cells counts. The slight changes in BALF parameters at 0.25 mg/m^3^ recovered and signs of lung clearance of the MWCNT were observed. No pathological changes were observed on the pleura. Neither increase in the number of micronucleated polychromatic erythrocytes nor increase in percent DNA damage were observed at any concentration.

**Conclusions:**

Lung inflammation characteristic of an overload with insoluble particles was observed after a 90-day exposure to 5.0 mg/m^3^ of Graphistrength^**©**^ C100. Clear signs of clearance and recovery were observed at 0.25 mg/m^3^. No genotoxicity was detected locally in lung and distally in bone marrow, liver and kidney. Therefore, Graphistrength^**©**^ C100 appears of low concern in term of local and systemic genotoxicity and a No-Observed Adverse Effect Concentration (NOAEC) of 0.25 mg/m^3^ (0.28 mg/m^3^ as actual concentration) was established for the repeated-dose toxicity.

**Electronic supplementary material:**

The online version of this article (doi:10.1186/s12989-015-0096-2) contains supplementary material, which is available to authorized users.

## Background

Multiwalled carbon nanotubes (MWCNT) are emerging new materials intended for use in aeronautics, automotive, electronics, and many other fields. The outstanding performance of MWCNT in applications like electrical conduction or mechanical improvements makes them valuable in the development of new light materials with improved properties [1, 2]. There is not just a single kind of MWCNT. There are diverse materials whose physico-chemical and toxicological properties depend on several factors: number of walls, diameter, length, shape (needle-like shape or flexible and contorted), states of agglomeration and aggregation, surface chemistry, metallic impurities, to name a few. Determination of the potential health effects of MWCNT has become a focus of attention in the scientific and regulatory community. The possible impact on the health of workers involved in manufacture and handling and the development of wide spread use including a number of consumer uses [3] prompted studies of various materials using different routes of exposure. The similarity in the shape and dimension of some kinds of MWCNT and asbestos was reported by Poland in 2008 [4]. Toxicology studies confirmed the induction of inflammation, granulomas and/or mesotheliomas by a long, thick and rigid (needle-like) type of MWCNT (MWCNT-7 from Mitsui, diameter 70-170 nm, length ca. 5 μm) after intraperitoneal (i.p.) injection to p53^+/-^ mice [5, 6] and Fischer 344/Brown Norway F1 hybrids rats [7], injection into the pleural space of C57Bl/6 mice [8], and intrascrotal injection to Fischer 344 rats [9]. Inhalation exposure of male B6C3F1 mice to MWCNT-7 for 15 days at a concentration of 5 mg/m^3^ promoted bronchioloalveolar adenoma and carcinoma induced by a single i.p. injection of methylcholanthrene [10]. MWCNT-7 was also found to induce oxidative DNA damages and gene mutations in the lung cells of mice after a single intratracheal (i.t.) instillation [11]. In contrast, no fibrotic lesions were observed [8] after the intrapleural injection to C57Bl/6 mice of short and straight (diameter 20-30 nm, length 0.5-2 μm, from Nanostructured & Amorphous Materials, Inc.) or curled/tangled (diameter ca. 15 nm, length 1-5 μm and diameter ca. 10 nm, length 5-20 μm, both from NanoLab Inc.) MWCNT. Moreover, no increased incidence of mesothelioma and other tumors was recorded by Muller *et al.* [12] in male Wistar rats two years after a single i.p. injection of MWCNT (diameter ca 10 nm, length 0.7 μm, from Namur University) with or without structural defects. No induction of malignant mesothelioma in the peritoneal cavity [13] was also observed when rats were followed for up to 3 years after two i.p. injections of a tangled form of MWCNT (diameter 15 nm, length 3 μm, from Showa Denko [7]). Therefore, it seems that the rigidity, diameter, length, surface properties and possibly contaminant metals are key factors when considering the potential for a carcinogenic effect of MWCNT [13]*.* In view of the lack of coherent evidence across the various distinct MWCNT, the IARC Monograph Working Group [14] specifically classified MWCNT-7 as possibly carcinogenic to humans (Group 2B) and the other types of MWCNT were categorized as not classifiable in respect to their carcinogenicity to humans (Group 3).

Graphistrength^**©**^ C100 is one of the industrial MWCNT referenced in the sponsorship program for the safety testing of nanomaterials by the Organization for Economic Cooperation and Development (OECD) [15]. There are a number of publications [16–25] reporting studies on Graphistrength^**©**^ C100 under the coded named NM 402 or JRCNM04002a, sample from the repository of the European Commission Joint Research Centre (EU-JRC) [26]. This EU-JRC Graphistrength^**©**^ C100 was produced by Arkema in a pilot production unit, whereas, the Graphistrength^**©**^ C100 used in the present 90-day study was from an industrial production unit. Nevertheless, these two units used the same process of synthesis and there are no significant physico-chemical differences between the products from both production units.

Graphistrength^**©**^ C100 is formed of large MWCNT agglomerates with a mean particle size of about 400 μm and contains a residual amount (<0.23 %) of small agglomerates (<15 μm) [27]. These small particles are comparable to those observed by R’mili *et al.* [28] in the atmosphere of our high safety laboratory dedicated to scientific experiments with MWCNT, indicating a possible inhalation exposure to these small particles. Therefore, conducting an inhalation subchronic toxicity study was judged to be a key feature in the safety assessment of Graphistrength^**©**^ C100. However, Graphistrength^**©**^ C100 does not contain sufficient quantities of these small agglomerates to directly provide the test material necessary for an experimental inhalation study [27]. Thus an aerosol generation procedure, as reported in the additional file 1, was developed in order to perform a valid study which fulfils the requirement of the inhalation specific OECD test guidelines [29]. The micronisation process is an enrichment of the small particle fraction and is allowing the worst case material to be used as expected by the regulatory authorities [30]. Another important criterion was ensuring that the administered aerosol has physico-chemical properties similar to the original material. After a careful evaluation, the defined technical conditions for the generation of Graphistrength^**©**^ C100 aerosols were assessed in a 5-day range finding inhalation toxicity study in rats with a 28-day recovery period. Then, a 90-day inhalation toxicity study was performed in rats. It included a 90-day recovery period and an evaluation of the pulmonary inflammation parameters. This subchronic study also provided the opportunity to perform a micronucleus assay on the bone marrow cells, as well as, a standard and a hOGG1-modified comet assay on the lung, liver and kidney cells of the exposed rats. The hOGG1-modified comet assay was chosen because it is more specific than the FPG (formamidopyrimidine glycosilase) comet assay for the identification of oxidative DNA damage [31]. Thus, the genotoxic potential was evaluated in the cells directly in contact with Graphistrength^**©**^ C100, and at a distance in case material was translocated from the lungs.

## Results

### Physico-chemical analysis

Graphistrength^**©**^ C100 used in the 5-day (batch no. 8287) and the 90-day (batch no. 110329-018) studies have respectively a median agglomerate size distribution of 376 and 418 μm, an ash content of about 8.6 and 8.2 %, an apparent density of 0.085 and 0.106 g/cm^3^, a specific surface area of 187 and 225.6 m^2^/g and metal contents (from the catalyst) of 3.2 and 3.0 % for Al and 2.7 and 2.7 % for Fe. MWCNT constituents of Graphistrength^**©**^ C100 have respectively 11 ± 3 and 12 ± 4 walls with an outer mean diameter of 11.8 and 12.1 nm, and a length of 1.05 ± 0.67 and 1.07 ± 1.10 μm. The surface to volume ratio of the material used for the 90-day study was 2.4 × 10^7^ m^-1^. The other physico-chemical data are presented in Additional file 1: Table S3.

The physico-chemical characterizations of Graphistrength^**©**^ C100 (batch no. 110329-018) after a 12-h milling under argon and after aerosol generation (samples collected at the exhaust of the elutriator just before the inhalation chamber) are detailed in the Additional file 1: Table S3 and Additional file 1: Figure S4 and showed minimal changes between the starting material and the ball milled and micronized samples.

### Characterization of the aerosol during exposures

The gravimetrically determined mean achieved aerosol concentrations of 0.066, 0.26 and 1.30 mg/m^3^ air during the 5-day exposure study were close to the targets of 0.05, 0.25 and 1.25 mg/m^3^ (Additional file 1: Table S4), respectively. Over the 90-day exposure, the mean achieved aerosol concentrations of 0.06, 0.28 and 4.84 mg/m^3^ air were also close to the target concentrations of 0.05, 0.25 and 5.0 mg/m^3^, respectively (Table 1).

**Table 1:** Target and achieved aerosol concentrations and particles size of Graphistrength^©^ C100. Temperature, relative humidity and oxygen concentration measured over the 90-day exposure period

Groups	Control	Low	Mid	High
Target aerosol concentrations (mg/m^3^ air)	0	0.05	0.25	5.0
Achieved aerosol concentrations (mg/m^3^ air)	-	0.06 ± 0.04	0.28 ± 0.06	4.84 ± 0.41
Mean mass median aerodynamic diameter (MMAD, μm) (gravimetric determination)	-	nd^a^	1.62 ± 0.39	2.30 ± 0.34
			(n = 5)	(n = 14)
Mean GSD (gravimetric determination)	-	nd^a^	4.67 ± 4.81	2.47 ± 0.26
Mean percentage of particles < 3 μm (gravimetric determination)	-	nd^a^	74.10 ± 14.00	63.49 ± 6.23
Count median aerodynamic diameter (CMAD, nm) (WPRS determination)	-	196.2 ± 54.7	231,5 ± 65.1	208.0 ± 62.0
		(n = 14)	(n = 14)	(n = 14)
Mean temperature (°C)	23.2 ± 0.9	23.3 ± 0.7	23.6 ± 0.7	23.8 ± 0.6
Mean relative humidity (%)	5.8 ± 1.5	6.1 ± 1.7	6.1 ± 1.7	6.3 ± 1.6
Mean oxygen concentration (%)	20.8 ± 0.0	20.8 ± 0.0	20.8 ± 0.0	20.8 ± 0.0

nd: not determined

^a^ at 0.05 mg/m^3^ due to the very low concentration, the particle size could not be determined by gravimetry at an air flow rate of 1 L/min. The aerosol concentrations at 0.05 and 0.25 mg/m^3^ were achieved by serial dilution with compressed, filtered, dry air of the 0.25 and 5.0 mg/m^3^ concentrations, respectively. Therefore, the MMAD and GSD at 0.05 mg/m^3^ are expected to be of the same order as at 0.25 mg/m^3^. This is also confirmed by particle size data from the 5-day study with sampling at an air flow rate of 9 L/min (see Additional file 1: Table S4 for details)

The mean mass median aerodynamic diameters (MMAD) and geometric standard deviations (GSD) by impactor/gravimetric determinations during the 5-day and 90-day exposures were within the target ranges (Additional file 1: Table S4 and Table 1). The count median aerodynamic diameters (CMAD) determined by Wide Range Particle Spectrometer^**©**^ (WPRS) analysis (Table 1) were similar between the exposed groups of the 90-day exposure study. All together these data showed that the generated aerosols were within the respirable range for rats (MMAD < 3 μm).

Temperature, relative humidity and oxygen parameters were consistent during both treatment periods (Additional file 1: Table S4 and Table 1, respectively). In addition, values for temperature and oxygen concentration were similar across all groups. Dried air was used for aerosol generation and, accordingly, the relative humidity values were below 8 % for all groups. Differences between the groups were considered to be negligible at this level. Therefore, the exposure conditions were considered to be satisfactory for this type of studies.

### Ante-mortem animal observations

#### 5-day exposure with a 28-day recovery period

All animals survived the 5-day exposure and 28-day recovery periods without showing clinical signs (data not shown). The food intake was similar across all groups during the study and there were no effects on body weight that were considered to be related to exposure to Graphistrength^**©**^ C100. Stagnation of body weight gain or marginal body weight loss was noted between day 1 and day 5 of treatment for all groups including controls. It is not unusual in inhalation studies and was considered to be due to the restraining of the animals in the tubes during the nose-only exposure procedure and not related to treatment with the test material. Normal body weight gain was observed during the 28-day recovery period across all groups (data not shown).

#### 90-day exposure with a 90-day recovery period

All animals survived the 90-day exposure and recovery periods. There were no test-item related clinical signs in any group. Hair loss, scabs, erythema and localized swelling were recorded. These signs are commonly seen in animals of this age and strain and are, therefore, considered to be incidental. No effects on food consumption were observed during the 90-day treatment. Increased food intake was recorded in male rats exposed to 0.05 and 5.0 mg/m^3^ during the first week of the 90-day recovery and several weeks thereafter. In addition increased food intake was recorded during the first two weeks of recovery in females exposed to 5.0 mg/m^3^ (data not shown). Slightly reduced body weight gains were seen in males and females exposed to 0.25 and 5.0 mg/m^3^ during several weeks of exposure. However, the mean body weights of these animals remained similar to the control group during the exposure period. At 0, 0.05, 0.25, and 5.0 mg/m^3^, the body weights (means ± SDs) were at the commencement of study (day 0): 266.8 ± 11.9 g, 269.7 ± 9.9 g, 268.5 ± 12.2 g and 270.4 ± 11.0 g in males (35 rats per group), respectively and 170.7 ± 19.1 g, 170.8 ± 16.0 g, 174.3 ± 19.4 g and 177.9 ± 22.7 g in females (35 per group), respectively. At the end of the 90-day exposure period, the respective body weights were 401.7 ± 26.9 g, 407.3 ± 35.9 g, 405.3 ± 37.9 g and 411.2 ± 29.4 g in males (30 per group) and 244.7 ± 21.9 g, 240.3 ± 20.3 g, 248.0 ± 25.4 g and 245.9 ± 25.3 g in females (30 per group). Increased body weight gains in males and females and body weights in males were observed during recovery in animals exposed to 5.0 mg/m^3^. At 0, 0.05, 0.25, and 5.0 mg/m^3^, the respective body weights (means ± SDs) at the end of the 90-day recovery period were 494.5 ± 44.1 g, 529.0 ± 53.5 g, 518.8 ± 55.0 g and 539.3 ± 40.6 g in males (20 per group) and 297.1 ± 33.9 g, 290.2 ± 30.0 g, 298.5 ± 39.8 g and 302.4 ± 45.5 g in females (20 per group). None of these changes were considered to be adverse effects.

There were no effects of exposure on grip strength, body temperature, landing foot splay and locomotors activity. There were no differences in blood pressure that were considered to be related to the exposure. High systolic/diastolic blood pressures (ca. 140/100 mmHg) were observed in one male of each control and treated groups. No effect was recorded during ophthalmoscopic examination (data not shown).

The statistically significant changes observed in hematological parameters are summarized in Table 2. An increase in relative and absolute neutrophil counts and a slight decrease of the relative (but not absolute) lymphocyte counts were recorded in males and females exposed to 5.0 mg/m^3^ at the end of the 90-day exposure and recovery periods. The other statistically significant changes (relative eosinophil counts, prothrombin time, and platelets) were all in the range of the historical control data (HCD) of the laboratory (Table 2), not-dose-related, observed only in one sex and not observed after the 90-day treatment free period and therefore were not considered to be treatment-related.

**Table 2:** Statistically significant changes^a^ in hematological parameters 24 h and 90 days after a 90-day exposure of male and female rats to Graphistrength^©^ C100

Concentration	Total leucocytes	Neutrophils	Eosinophils	Lymphocytes	Prothrombin time	Platelets
(mg/m^3^)	G/L	rel. 1 (G/l)	rel. 1 (G/l)	rel. 1 (G/l)	rel. 1 (sec)	G/l
**MALE**						
HCD ^b, c^	3.74 - 9.53	0.119 - 0.339	0.01 - 0.035	0.611 - 0.842	0.70 – 0.97	708 - 1168
		(0.66 - 2.29)	(0.05 - 0.22)	(2.59 - 7.39)	(14.4 - 29.9)	
24 h post exposure						
0	6.43	0.197 (1.28)	0.009 (0.06)	0.742 (4.78)	0.73 (23.4)	879
0.05	7.01	0.220 (1.79)	0.013 (0.10)	0.716 (4.79)	0.73 (23.4)	973
0.25	7.30	0.211 (1.71)	0.013* (0.10)	0.727 (5.15)	0.76 (22.8)	972
5.0	8.13	0.327** (2.63**)	0.015* (0.12)	0.608** (5.05)	0.77* (22.5)	1003*
90 days post exposure						
0	7.07	0.237 (1.79)	0.012 (0.09)	0.707 (4.88)	0.85 (23.7)	903
0.05	7.47	0.223 (1.62)	0.014 (0.11)	0.716 (5.44)	0.86 (23.4)	914
0.25	7.14	0.283 (2.23)	0.015 (0.11)	0.664 (4.55)	0.85 (23.7)	822
5.0	8.23	0.374* (2.96**)	0.015 (0.12)	0.577* (4.84)	0.89 (22.8)	901
**FEMALE**						
HCD^b, c^	1.91 - 6.14	0.099 - 0.343	0.009 - 0.045	0.598 - 0.860	0.70 – 0.98	723 - 1235
		(0.34-1.31)	(0.03 - 0.15)	(1.30 - 4.86)	(13.5 - 38.2)	
24 h post exposure						
0	4.07	0.146 (0.61)	0.011 (0.04)	0.809 (3.29)	0.73 (23.5)	1144
0.05	4.05	0.152 (0.61)	0.006 (0.04)	0.808 (3.27)	0.75 (23.0)	1087
0.25	3.69	0.170 (0.62)	0.016 (0.06)	0.779 (2.87)	0.79** (22.2**)	1042
5.0	4.96	0.261** (1.21**)	0.012 (0.06)	0.698** (3.54)	0.77 (22.6)	1099
90 days post exposure						
0	4.17	0.175 (0.67)	0.017 (0.06)	0.772 (3.27)	0.87 (23.2)	883
0.05	3.75	0.220 (0.84)	0.019 (0.07)	0.729 (2.71)	0.88 (22.8)	933
0.25	3.32	0.238* (0.74)	0.018 (0.05)	0.714 (2.40)	0.86 (23.5)	841
5.0	4.90	0.382** (1.81**)	0.013 (0.07)	0.564** (2.85)	0.89 (22.7)	914

* *p* < 0.05, ** *p* < 0.01; statistical significant differences to controls

^a^ No statistically significant changes were observed at any concentrations and time-points on red blood cell, hemoglobin, hematocrit, mean corpuscular volume, mean corpuscular hemoglobin, mean corpuscular hemoglobin concentration, reticulocytes, basophils, monocytes and partial thromboplastine time

^b^ HCD Historical control data, 95 % tolerance limits

^c^ Changes statistically significant outside the HCD are in italic characters

The statistically significant changes of the blood chemistry parameters are summarized in Table 3. Increased potassium values were recorded in males exposed to 5.0 mg/m^3^ (9 %) and in all treated groups of females (15, 21 and 11 % at 0.05, 0.25 and 5.0 mg/m^3^, respectively) at the end of the treatment period but not after the 90-day treatment free period. Considering the low magnitude of this hyperkalemia and the variability of the potassium levels in the rats [32], these changes were not considered to be treatment related and/or adverse. The other statistically significant changes (creatinine, triglycerides, sodium, chloride, calcium, and proteins) were not considered to be treatment-related as the values were all in the range of the HCD data (Table 3), not dose-related, observed only in one sex, and not correlated with histological findings.

**Table 3:** Statistically significant changes^a^ in blood chemistry parameters 24 h and 90 days after a 90-day exposure of male and female rats to Graphistrength^©^ C100

Concentration (mg/m^3^)	Creatinine μmol/l	Triglycerides mmol/l	Sodium mmol/l	Potassium mmol/l	Chloride mmol/l	Calcium mmol/l	Total protein g/l
**MALE**							
HCD^b, c^	21.9 - 35.0	0.20 - 1.08	138. 5- 149.2	3.22 - 4.47	99.9 - 109.2	2.55 - 2.97	62.10 - 73.54
24 h post exposure							
0	23.7	0.48	142.8	4.13	102.3	2.68	66.15
0.05	24.6	0.38*	142.9	4.20	102.1	2.67	65.39
0.25	23.6	0.37*	143.9	4.25	103.0	2.69	67.02
5.0	22.4	0.41	144.2	4.52**	103.0	2.69	66.97
90 days post exposure							
0	27.9	0.77	145.5	4.52	103.4	2.75	69.08
0.05	25.5*	0.68	145.7	4.59	103.2	2.77	68.42
0.25	24.4**	0.75	145.9	4.58	104.4	2.71	67.41
5.0	26.3	0.79	145.8**	4.73	104.5	2.74-	68.24
**FEMALE**							
HCD^b, c^	25.0 - 41.7	0.18 - 0.57	137.8 - 147.8	2.73 - 3.90	100.6 - 110.3	2.53 - 2.99	63.62 - 79.36
24 h post exposure							
0	30.4	0.32	143.5	3.43	102.0	2.75	72.27
0.05	27.7	0.31	144.8	3.95**	103.8	2.76	71.50
0.25	28.3	0.35	144.0	4.17**	103.4	2.80	72.87
5.0	27.3*	0.30	145.8	3.81*	105.3**	2.72	69.27*
90 days post exposure							
0	28.8	0.72	143.5	3.40	101.7	2.78	77.11
0.05	31.9	0.60	146.4**	3.41	104.1	2.76	74.74
0.25	30.1	0.54*	144.9	3.21	101.9	2.72*	74.78
5.0	30.6	0.47**	145.1	3.54	104.0	2.74	72.29**

* *p* < 0.05, ** *p* < 0.01; statistical significant differences to controls

^a^ No statistically significant changes were observed at any concentrations and time-points on glucose, urea, bilirubin, cholesterol, triglycerides, phospholipids, aspartate aminotransferase (ASAT), alanine aminotransferase (ALAT), lactate dehydrogenase (LDH), alkaline phosphatase (ALP), gamma-glutamyltransferase (GGT), creatine kinase (CK), phosphorus, albumin and globulin

^b^ HCD Historical control data, 95 % tolerance limits

^c^ Changes statistically significant outside the HCD are in italic characters

Urinalysis and estrus cycles parameters were unremarkable (data not shown).

### Post-mortem animal observations

#### Bronchoalveolar lavage fluid (BALF) analysis

**Fig. 1:**
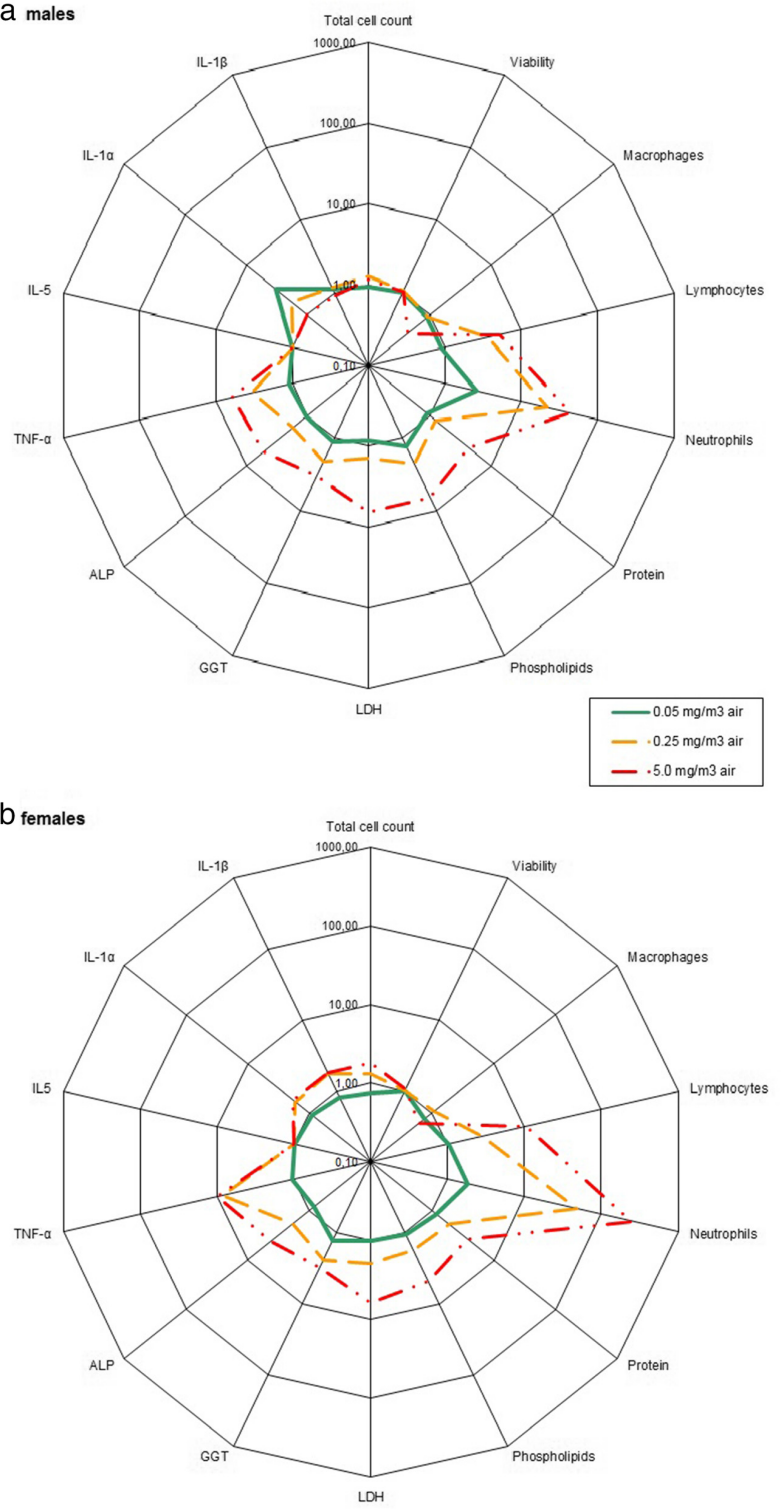
BALF parameters of male (**a**) and female (**b**) rats 24 h after a 90-day exposure to Graphistrength^©^ C100. Changes are shown as x-fold differences compared to controls using a logarithmic scaling. Abbreviations: ALP: alkaline phosphatase, GGT: γ-glutamyltransferase, LDH: lactate dehydrogenase, IL-1β: interleukin 1β, TNF-α: Tumor Necrosis Factor α

**Fig. 2:**
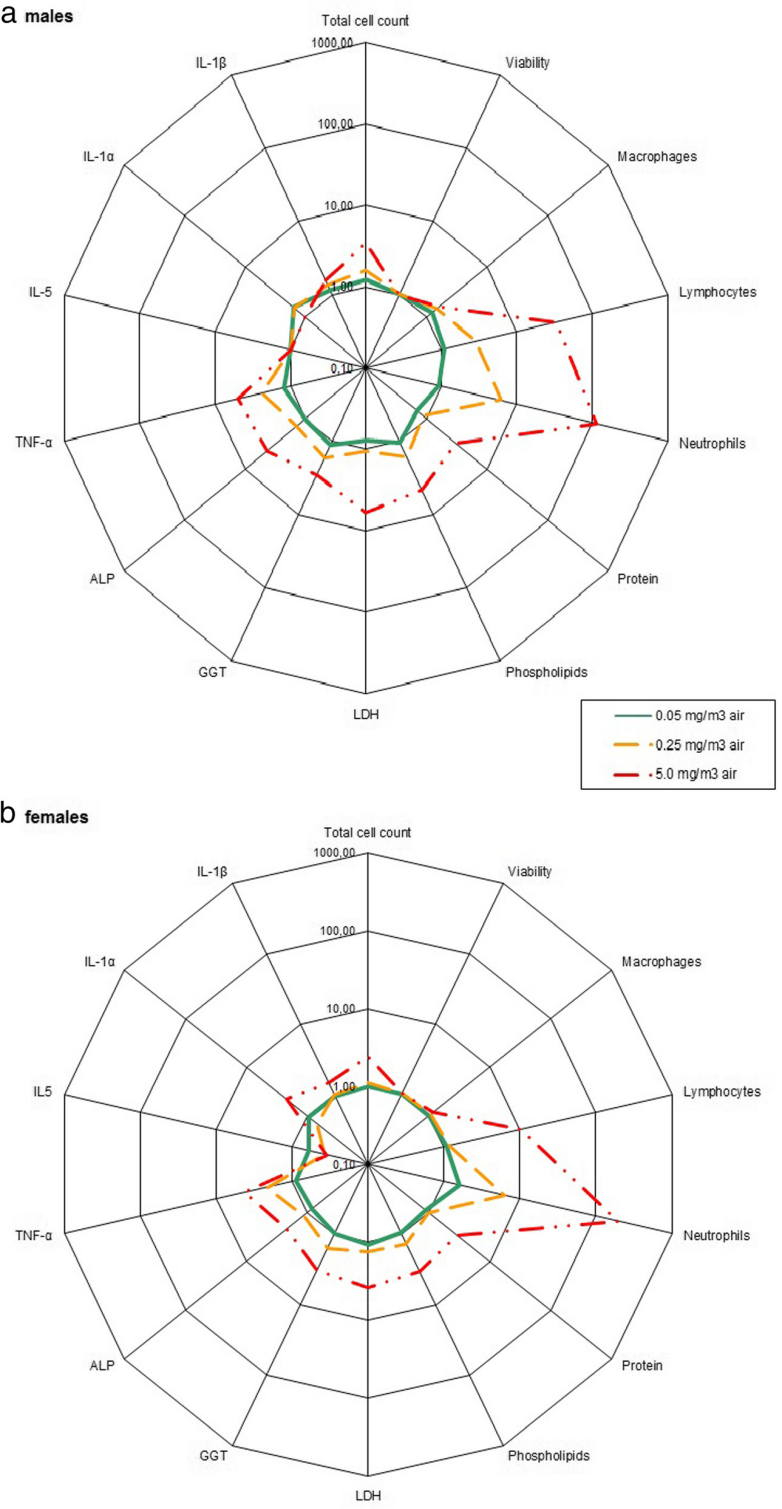
BALF parameters of male (**a**) and female (**b**) rats 90 days after a 90-day exposure to Graphistrength^©^ C100. Changes are shown as x-fold differences compared to controls using a logarithmic scaling. Abbreviations: ALP: alkaline phosphatase, GGT: γ-glutamyltransferase, LDH: lactate dehydrogenase, IL-1β: interleukin 1β, TNF-α: Tumor Necrosis Factor α

Detailed results of cellular, biochemical and cytokines measurements are displayed in Additional file 1: Tables S5 and Additional file 1: Table S6 for the 5-day study and Tables 4, 5, 6 for the 90-day study. As the methods used to collect of the BALF were slightly different between the 5-day (use of the full lung) and the 90-day (use of only the left lobe) studies, and to also allow a comparison with the recovery data, changes in BALF parameters presented in Additional file 1: Figure S5 (5-day study) and Figs. 1 and 2 (90-day study) were normalized relative to the time-matched, concurrent control group.

**Table 4:** Cell analysis in the BALF of male and female rats 24 h and 90 days after a 90-day exposure to Graphistrength^©^ C100

Concentration (mg/m^3^)	Total cell Count	Viability	Macrophages	Eosinophils	Lymphocytes	Neutrophils	Epithelial cells
	10^6^	%	%	%	%	%	
**MALE**							
24 h post exposure							
0	1.74	84.40	93.9	0.0	4.4	1.5	0.1
0.05	1.62	84.65	91.8	0.0	5.1	3.1	0.0
0.25	2.24	89.00	64.8**	0.0	9.7*	25.2**	0.3
5.0	2.01	88.00	31.5**	0.0	12.8**	55.6**	0.1
90 days post exposure							
0	1.80	90.10	96.5	0.0	2.1	1.2	0.2
0.05	2.21	91.35	96.9	0.0	1.9	0.9	0.3
0.25	2.85*	91.35	91.2	0.1	3.8	4.7	0.3
5.0	6.23**	89.86	44.6**	0.0	20.4**	39.8**	0.3
**FEMALE**							
24 h post exposure							
0	1.67	91.80	96.9	0.0	2.5	0.4	0.1
0.05	1.27	92.55	94.5	0.0	4.4	1.0	0.1
0.25	2.16	92.55	80.9**	0.0	4.1	15.0**	0.0
5.0	2.98**	95.50	34.7**	0.0	11.5**	53.8**	0.0
90 days post exposure							
0	1.67	86.39	96.1	0.0	3.1	0.5	0.2
0.05	1.66	85.40	95.8	0.0	3.4	0.8	0.0
0.25	1.86	87.55	93.9	0.0	3.3	2.8	0.0
5.0	4.02**	88.80	46.7**	0.0	13.0**	40.1**	0.0

* *p* < 0.05, ** *p* < 0.01; statistical significant differences to controls

#### 5-day exposure with a 28-day recovery period

Twenty-four hours post exposure, the differential cell count in BALF revealed statistically significantly increased polymorphonuclear neutrophils (PMN) levels at 1.25 mg/m^3^ (x9 compare to control group) as detailed in Additional file 1: Table S5 and Additional file 1: Figure S5 panel A. After the 28-day recovery period the PMN value was similar to controls (Additional file 1: Table S5 and Additional file 1: Figure S5 panel B). Twenty-four hours post exposure, the macrophages in all treated groups contained phagocytized test material with a dose-related increased incidence (Additional file 1: Table S5). At the end of the recovery period, the incidence significantly decreased at all concentrations (Additional file 1: Table S5).

Biochemical analysis of the BALF 24 h post-exposure resulted in statistically significantly increased γ-glutamyltransferase (GGT) levels at 1.25 mg/m^3^ (x3.5) and increased protein values at 0.25 (x23) and 1.25 (x20) mg/m^3^ (Additional file 1: Table S6 and Additional file 1: Figure S5 panel A). After the 28-day recovery period, differences to controls diminished and statistical significance was only observed for changes in protein values at 0.25 (x3) and 1.25 (x3.5) mg/m^3^ (Additional file 1: Table S6, Additional file 1: Figure S5 panel B). All other changes between exposed and control groups and between the 24-h-and 28-day sacrifice times are just biological variations.

#### 90-day exposure with a 90-day recovery period

Twenty-four hours post exposure, there were no changes in BALF total cell count and viability in males (Table 4, Fig. 1 panel a). Nevertheless, it must be mentioned that these 2 parameters have been determined in only 4 out of 10 males rats exposed at 5.0 mg/m^3^ due to the large amount of black particles and MWCNT-laden BAL cells in the cellular suspension. In females, the total cell count was increased at 5.0 mg/m^3^ (+78 %) while viability did not show any differences (Table 4, Fig. 1 panel b. The cytodifferentiation of BALF cells (Table 4, Fig. 1) showed decreases in macrophages and increases in neutrophils and lymphocytes in males and females exposed to 0.25 and 5.0 mg/m^3^, respectively.

Statistically significant increases in protein (x4.3 and x3.7), phospholipids (x6.6 and x4.8), LDH (x6.6 and x6.2), GGT (x3.3 and x 3.1) and ALP (x5.2 and 4.1) were recorded in males and females exposed at 5.0 mg/m^3^, respectively (Table 5, Fig. 1). In addition phospholipids in males (*x*2.2) and GGT in males (*x*2.1) and females (*x*2.4) were also increased at 0.25 mg/m^3^.

**Table 5:** Biochemistry parameters in the BALF of male and female rats 24 h and 90 days after a 90-day exposure to Graphistrength^©^ C100

Concentration (mg/m^3^)	Phospholipids mmol/L	LDH U/L	ALP U/L	GGT U/L	Protein mg/L
**MALE**					
24 h post exposure					
0	0.16	196.8	37.6	5.9	92.7
0.05	0.21	168.6	38.8	6.7	84.2
0.25	0.36*	275.4	64.7	12.5**	115.4
5.0	1.05**	1306.2**	195.4**	19.5**	395.0**
90 days post exposure					
0	0.26	225.7	76.4	5.6	115.4
0.05	0.27	174.8	78.2	6.3	79.8
0.25	0.42	237.2	108.5	9.5	102.3
5.0	1.18**	1353.2**	330.3**	16.1**	347.4**
**FEMALE**					
24 h post exposure					
0	0.17	163.6	34.9	5.1	78.8
0.05	0.18	163.0	28.8	6.6	91.2
0.25	0.30	312.7	63.4	12.5**	146.4
5.0	0.82**	1020.2**	144.1**	15.9**	292.6**
90 days post exposure					
0	0.23	147.9	55.2	5.0	103.5
0.05	0.22	159.0	45.3	4.8	90.8
0.25	0.31	194.5	63.0	7.8	103.5
5.0	0.76**	566.2**	117.8**	15.8**	300.2**

* *p* < 0.05, ** *p* < 0.01; statistical significant differences to controls

A statistically and biologically significant increase (values higher than mean + 2 SD of the corresponding control group) of tumor necrosis factor alpha (TNF-α) was observed in BALF of male and female rats exposed to 0.25 (x3.4 and x8.5, respectively) and 5.0 mg/m^3^ (x6.1 and x10, respectively) and of IL-1β levels (x1.8) in females exposed to 5.0 mg/m^3^ (Table 6, Fig. 1). Detectable levels of IL-5 and IL-1α (data not shown) were measured in some single animals (including controls) without a dose-response relationship.

**Table 6:** Cytokines parameters in the BALF of male and female rats 24 h and 90 days after a 90-day exposure to Graphistrength^©^ C100

Mean ± sd (pg/mL) #	IL-1β	IL-5	TNF-α	IL-1 α
**MALE**				
24 h post exposure				
0 mg/m^3^	11.52 ± 6.43	0 ± 0.0	1.34 ± 0.60	3.38 ± 6.40
0.05 mg/m^3^	12.83 ± 9.11	0 ± 0.0	1.47 ± 0.84	11.31 ± 11.70
0.25 mg/m^3^	13.43 ± 5.96	0 ± 0.0	4.56 ± 1.55**	5.99 ± 6.82
5.0 mg/m^3^	10.60 ± 2.58	0 ± 0.0	*8.17 * ± 2.66**	0 ± 0.0
90 days post exposure				
0 mg/m^3^	15.68 ± 5.65	2.30 ± 4.87	1.83 ± 0.75	6.46 ± 4.20
0.05 mg/m^3^	18.40 ± 3.69	7.12 ± 4.43*	2.21 ± 2.25	10.20 ± 8.39
0.25 mg/m^3^	21.38 ± 6.63*	12.38 ± 9.33**	*4.46 * ± 1.54**	9.82 ± 8.16
5.0 mg/m^3^	24.64 ± 7.28**	4.63 ± 6.38	*9.03 * ± 2.28**	*21.74 * ± 24.45*
**FEMALE**				
24 h post exposure				
0 mg/m^3^	7.74 ± 2.90	1.34 ± 1.78	0.77 ± 0.20	7.15 ± 9.35
0.05 mg/m^3^	6.31 ± 3.24	0.78 ± 1.27	0.80 ± 0.37	6.44 ± 10.70
0.25 mg/m^3^	13.48 ± 5.58	0.06 ± 0.18	*6.53 * ± 7.33**	11.94 ± 11.61
5.0 mg/m^3^	13.81 ± 7.47**	0.57 ± 1.81	*7.68 * ± 1.35**	13.16 ± 17.70
90 days post exposure				
0 mg/m^3^	16.83 ± 4.57	13.41 ± 11.01	2.12 ± 0.85	9.94 ± 6.86
0.05 mg/m^3^	15.63 ± 4.22	8.00 ± 3.54	1.89 ± 0.66	9.52 ± 10.59
0.25 mg/m^3^	16.42 ± 3.66	4.98 ± 5.00	*4.54 * ± 1.33**	6.75 ± 8.24
5.0 mg/m^3^	24.57 ± 5.42**	1.46 ± 3.60	*11.16 * ± 4.91**	4.49 ± 6.54

* *p* < 0.05, ** *p* < 0.01; statistical significant differences to controls

# mean value higher than mean + 2sd of the corresponding control group are in italic characters

Ninety days post-exposure, there was an increase in the total cell counts in male and female rats exposed to 5.0 mg/m^3^ (x3.5 and *x*2.4, respectively) but viabilities were not affected (Table 4, Fig. 2). Differential counts were still changed with a similar intensity as 24-h post-exposure. In males exposed to 0.25 mg/m^3^, an increase of the total cell count (+58 %) was observed, but it was not present 24 h post-exposure. As this increase was not associated with changes in differential counts, biochemistry parameters, and lung pathological effects, it was thought to be of no toxicological significance and related to the clearance process.

The changes in the biochemical parameters (Table 5, Fig. 2) and cytokines levels (Table 6, Fig. 2) observed at 5.0 mg/m^3^ were of similar intensity as at 24-h post-exposure. No change of the biochemical parameters was observed at 0.05 and 0.25 mg/m^3^ except a persistent increase of TNF-α levels in males and females exposed at 0.25 mg/m^3^ (*x*2.4 and *x*2.1, respectively) related to the residual amount of black particles in the lungs.

### Organs weights and macroscopic examination

#### 5-day exposure with a 28-day recovery period

There were no macroscopic lesions that could be attributed to treatment with Graphistrength^**©**^ C100. No change was observed in lung weight at either sacrifice times (data not shown). The absolute kidney weight was statistically significantly reduced just after the 5-day exposure in males exposed to 0.25 and 1.25 mg/m^3^ (1.71 and 1.77 g *vs.* 2.00 g in control, respectively, p < 0.01) as well as the kidney to body weight ratio at 0.25 mg/m^3^ (0.56 % *vs.* 0.61 % in control, *p* < 0.05). However, in the absence of histological correlates, effects in females and after the 28-day recovery period, the kidney weight change was not considered to be treatment-related. There were no further effects on organ weights which were considered to be possibly related to treatment (data not shown).

#### 90-day exposure with a 90-day recovery period

Black brown foci in the lung and black brown discoloration of the bronchial lymph nodes were recorded in all or most animals 24 h and 90 days after 90 days of exposure to 5.0 mg/m^3^. No test item-related macroscopic findings were observed in animals exposed to 0.05 and 0.25 mg/m^3^.

Twenty-hour hours post-exposure, absolute and relative (to body weight) lung weights were increased in males (+47 and +50 %, respectively) and females (+45 and +50 %, respectively) exposed to 5.0 mg/m^3^ (Table 7). Lung weight (absolute and relative) were still increased in males (+62 and +53 %, respectively) and females (+45 and +36 %, respectively) exposed to 5.0 mg/m^3^ after 90 days of recovery (Table 7). All other statistically significant changes in organ weights were incidental and not considered to be treatment-related (data not shown).

**Table 7:** Lung weights of male and female rats 24 h and 90 days after a 90-day exposure to Graphistrength^©^ C100

Mean ± sd	0 mg/m^3^	0.05 mg/m^3^	0.25 mg/m^3^	5.0 mg/m^3^
**MALE**				
24 h post exposure				
Absolute weight (g)	1.50 ± 0.06	1.48 ± 0.14	1.63 ± 0.13	2.21 ± 0.29**
% body weight	0.38 ± 0.03	0.39 ± 0.02	0.41 ± 0.02	0.57 ± 0.05**
90 days post exposure				
Absolute weight (g)	1.49 ± 0.08	1.58 ± 0.16	1.56 ± 0.07	2.42 ± 0.30**
% body weight	0.30 ± 0.02	0.32 ± 0.02	0.31 ± 0.03	0.46 ± 0.05**
**FEMALE**				
24 h post exposure				
Absolute weight (g)	1.19 ± 0.10	1.05 ± 0.07*	1.26 ± 0.09	1.73 ± 0.18**
% body weight	0.48 ± 0.04	0.46 ± 0.03	0.51 ± 0.04	0.72 ± 0.04**
90 days post exposure				
Absolute weight (g)	1.18 ± 0.14	1.20 ± 0.09	1.27 ± 0.14	1.73 ± 0.12**
% body weight	0.38 ± 0.04	0.42 ± 0.03	0.44 ± 0.05**	0.64 ± 0.06**

*/**: Dunnett-test based on pooled variance significant <0.05 (*) or <0.01 (**)

### Microscopic examination

#### 5-day exposure with a 28-day recovery period

Findings observed in the lungs are summarized in Additional file 1: Table S7 and typical lesions are shown in Additional file 1: Figure S6. Twenty-four hours post exposure, hypertrophy of the bronchial and bronchiolar epithelial cells (Additional file 1: Figure S6 panel B) and an increased severity of the infiltration of macrophages were noted in several animals exposed to 1.25 mg/m^3^. Black inclusions in the cytoplasm of the infiltrated macrophages (Additional file 1: Figure S6 panel C) were observed in a concentration-dependent manner in all exposed animals confirming the observations done in the BALF.

At the end of the 28-day recovery period, all these findings were still present at a similar level, however, the incidence and the severity of hypertrophy of the bronchial and bronchiolar epithelial cells tended to be lower (only 4 animals affected, all with a minimal grade) (Additional file 1: Figure S6 panel D). The other microscopic findings were incidental lesions or within the range of normal background lesions for animals of this strain and age.

#### 90-day exposure with a 90-day recovery period

**Fig. 3:**
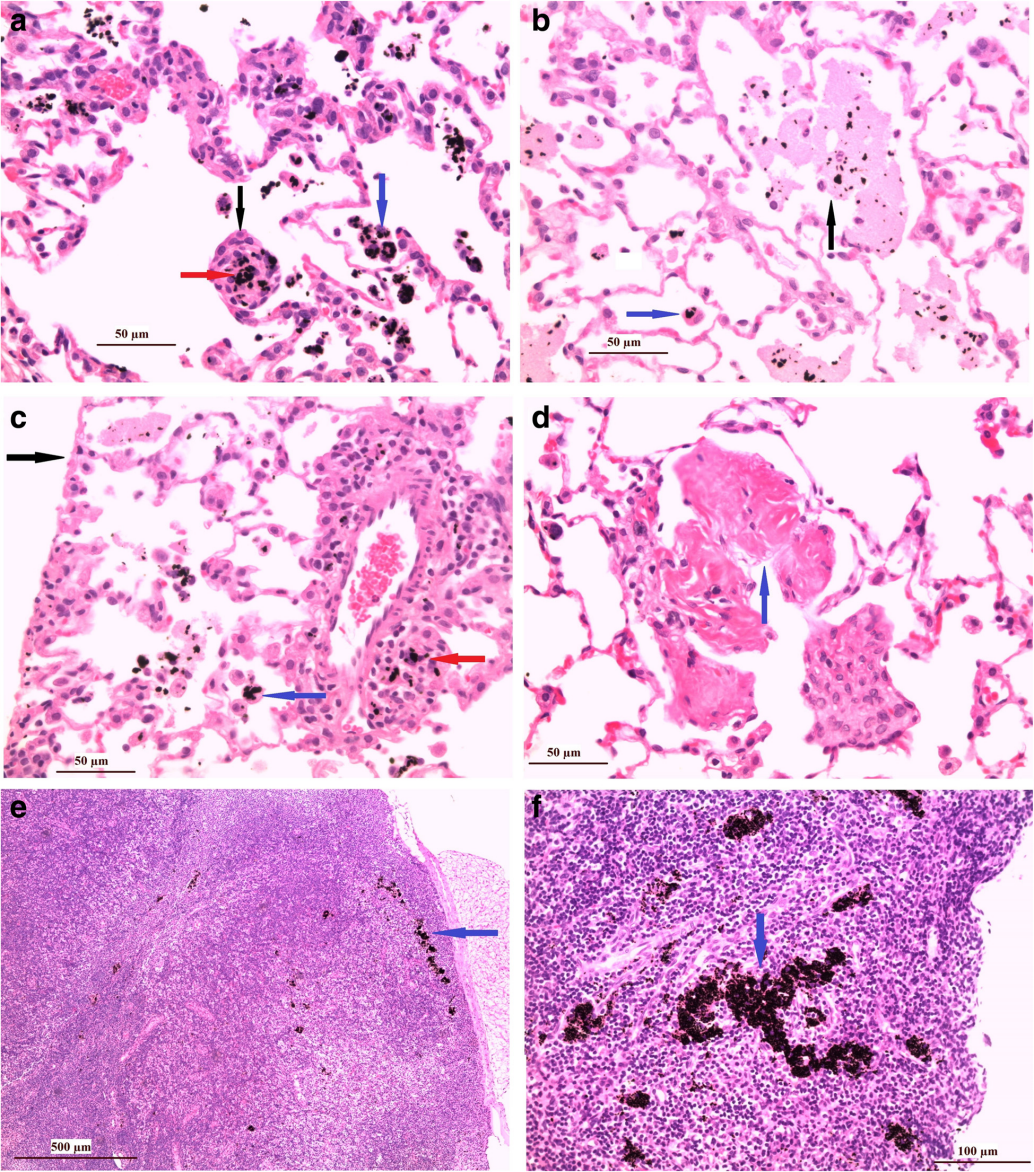
Microscopic appearance of lungs around terminal bronchiole and tracheochonchial lymph nodes 24 h or 90 days after a 90-day exposure to 5.0 mg/m^3^ of Graphistrength^©^ C100. **a** End of exposure. Presence of black particles within the alveolar macrophages (blue arrow) and tissue macrophages (red arrow). Note the interstitial inflammation around the alveolar duct with macrophages arranged as a small nodule-like structure (black arrow), forming concentric layers around black particles. **b** End of exposure. Presence of black particles within alveolar macrophages (blue arrow) or free within the alveolar lumen, admixed with the eosinophilic material (black arrow). **c** End of exposure. Presence of black particles within the alveolar macrophages (blue arrow) and tissue macrophages (red arrow) around a blood vessel. Note the intact pleura (black arrow). **d** End of recovery. Presence of interstitial collagen fibers protruding within the alveolar lumen (blue arrow) (**e**) End of exposure. Presence of black particles (blue arrow) in tracheobronchial lymph node associated with increased lymphocytes in the cortex/paracortex. **f** End of recovery. Tracheobronchial lymph node with large deposit of black particles (blue arrow). Original lens magnification: (**a**), (**b**), (**c**) and (**d**): 40-fold; (**e**): 6.3-fold; (**f**): 25-fold

Results of the microscopic examinations are presented in Tables 8, 9 and 10 for lungs, tracheobronchial lymph nodes and nasal cavity and larynx, respectively. Typical lesions in lungs and tracheobronchial lymph nodes are shown in Fig. 3.

**Table 8:** Microscopic findings in the lungs of male and female rats 24 h and 90 days after a 90-day exposure to Graphistrength^©^ C100

	Males				Females			
Target concentration (mg/m^3^)	0	0.05	0.25	5.0	0	0.05	0.25	5.0
Number examined	10/10	10/10	10/10	10/10	10/10	10/10	10/10	10/10
(24 h/90 days)								
**Black particle deposition**								
trace	-	-/10	-	-	-	-/10	-	-
minimal	-	9/-	-/10	-	-	10/-	2/10	-
slight	-	-	10/-	-	-	-	8/-	-
moderate	-	-	-	-/2	-	-	-	4/3
marked	-	-	-	10/8	-	-	-	6/7
Mean severity^a^	-	0.9/0.5	2.0/1.0	4.0/3.8	-	1.0/0.5	1.8/1.0	3.6/3.7
**Alveolar macrophages**								
minimal	-	-	8/10	-/1	-	-	9/10	-
slight	-	-	-	-/3	-	-	-	1/8
moderate	-	-	-	10/6	-	-	-	9/2
Mean severity^a^	-	0.0	0.8/1.0	3.0/2.5	-	-	0.9/1.0	2.9/2.2
**Alveolar eosinophilic material**								
minimal	-	-	-	6/8	-	-	-	4/4
slight	-	-	-	2/2	-	-	-	4/5
moderate	-	-	-	1/-	-	-	-	1/-
Mean severity^a^	-	-	-	1.3/1.2	-	-	-	1.5/1.4
**Alveolar granulocyte infiltration**								
minimal	-	-	-	10/8	-	-	-	10/7
Mean severity^a^	-	-	-	1.0/0.8	-	-	-	1.0/0.7
**Interstitial inflammation**								
minimal	-	-	-	-/1	-	-	-	4/5
slight	-	-	-	10/9	-	-	-	6/5
Mean severity^a^	-	-	-	2.0/1.9	-	-	-	1.6/1.5
**Bronchiolar cell hypertrophy/hyperplasia**								
minimal	-	-	-	10/-	-	-	-	10/-
Mean severity^a^	-	-	-	1.0/-	-	-	-	1.0/-
**Focal/multifocal collagen depositions, alveolar septa**								
minimal	-	-	-	-	-	-	-	-/2
slight	-	-	-	-/2	-	-	-	-
Mean severity^a^	-	-	-	-/0.4	-	-	-	-/0.2
**Increased lymphocytes, BALT** ^**b**^								
minimal	-	-	-	2/-	-	-	-	2/-
slight	-	-	-	1/-	-	-	-	-
Mean severity	-	-	-	0.4/-	-	-	-	0.2/-

Histopathological findings were graded in severity using a five point system of minimal (grade 1), slight (grade 2), moderate (grade 3), marked (grade 4) or severe (grade 5). An additional grading unit, trace (grade 0.5), was used to describe a trace amount of black particle deposition in the lungs

^a^: mean severity is ∑ number of animals x severity / number of examined organs in the group

^*b*^bronchus associated lymphoid tissue

- : no animal affected

**Table 9:** Microscopic findings in the tracheobronchial lymph nodes of male and female rats 24 h and 90 days after a 90-day exposure to Graphistrength^©^ C100

	Males				Females			
Target concentration (mg/m^3^)	0	0.05	0.25	5.0	0	0.05	0.25	5.0
Number examined	10/10	10/8	10/9	10/10	10/8	9/10	10/10	10/10
(24 h/90 days)								
**Black particle deposition**								
minimal	-	-	8/5	-	-	-	5/3	1
slight	-	-	-/2	6/3	-	-	-	7/1
moderate	-	-	-	4/7	-	-	-	1/9
Mean severity^a^	-	-	0.8/0.9	2.4/2.7	-	-	0.5/0.3	1.8/2.9
**Increased lymphocytes, cortex/paracortex**								
minimal	-	-	3/-	4/7	-	-	1/-	3/6
slight	-	-	-	3/2	-	-	-	7/-
moderate	-	-	-	3/-	-	-	-	-
Mean severity^a^	-	-	0.3/-	1.9/1.1	-	-	0.1/-	1.7/0.6
**Endothelial vacuolation, high endothelial venule**								
minimal	-	-	1/-	1/2	-	-	-	2/5
slight	-	-	1/-	7/1	-	-	-	5/-
Mean severity^a^	-	-	0.3/-	1.5/0.4	-	-	-	1.2/0.5

Histopathological findings were graded in severity using a five point system of minimal (grade 1), slight (grade 2), moderate (grade 3), marked (grade 4) or severe (grade 5)^a^: mean severity is ∑ number of animals x severity / number of examined organs in the group

^a^: mean severity is ∑ number of animals x severity / number of examined organs in the group - : no animal affected

**Table 10:** Microscopic findings in the nasal cavity and larynx of male and female rats 24 h and 90 days after a 90-day exposure to Graphistrength^©^ C100

	Males				Females			
Target concentration (mg/m^3^)	0	0.05	0.25	5.0	0	0.05	0.25	5.0
Number examined	10/10	10/0	10/10	10/10	10/10	10/0	10/10	10/10
(24 h/90 days)								
**Nasal cavity epithelium, eosinophilic globules**								
minimal	4/2	-	2/3	2/6	-/3	-	3/2	1/4
slight	-/1	-	-	3/2	-	-	-	6/2
moderate	-	-	-	5/2	-	-	-	2/3
Mean severity^a^	0.4/0.4	-	0.2/0.3	2.3/1.6	-/0.3	-	0.3/0.2	1.9/1.7
**Larynx, squamous metaplasia**								
minimal	-	-	2/-	4/-	-	-	-	5/1
Mean severity^a^	-	-	0.2/-	0.4/-	-	-	-	0.5/0.1

Histopathological findings were graded in severity using a five point system of minimal (grade 1), slight (grade 2), moderate (grade 3), marked (grade 4) or severe (grade 5)^a^: mean severity is ∑ number of animals x severity / number of examined organs in the group

^a^: mean severity is ∑ number of animals x severity / number of examined organs in the group - : no animal affected

Twenty-four hours post-exposure to Graphistrength^**©**^ C100, minimal to marked concentration-related deposition of black particles were recorded in the lungs of all exposed rats (Table 8, Fig. 3 panel a; for comparison a picture of the lung of a control rat is displayed in Additional file 1: Figure S6 panel A). After 90 days of recovery, the mean severity decreased at 0.05 and 0.25 mg/m^3^, indicating partial clearance of the black particles at these two lower concentrations; however, at 5.0 mg/m^3^, the mean severity score were overall similar indicating incomplete clearance during this timeframe in these lungs overloaded with Graphistrength^**©**^ C100 particles. At both sacrifice times, this deposition was associated with minimal to moderate concentration-related infiltration of alveolar macrophages at 0.25 and 5.0 mg/m^3^ and with alveolar eosinophilic material (considered to be the result of macrophages membrane cell rupture, Fig. 3 panel b), minimal infiltration of neutrophils in the alveoli (Fig. 3 panel c), and minimal to slight interstitial inflammation (Fig. 3 panel a) at 5.0 mg/m^3^ only. Noteworthy differences at the end of the 90-day recovery period were the presence of minimal increased interstitial collagen fibers (Fig. 3 panel d) in 3 males and 2 females exposed to 5.0 mg/m^3^ while the minimal bronchiolar cell hypertrophy/hyperplasia and increased lymphocytes in the bronchus associated lymphoid tissue (BALT) occasionally observed just post-exposure were not recorded anymore. Minimal deposition of black particles was also seen at the tracheal bifurcation in some animals exposed at 5.0 mg/m^3^ at both sacrifice times.

Minimal to moderate dose-related deposition of black particles in the tracheobronchial lymph nodes was the histological correlate of the black discoloration recorded at necropsy. This finding was recorded 24 h post-exposure in animals exposed to 0.25 and 5.0 mg/m^3^ (Table 9, Fig. 3 panel e) and was associated with increased lymphocytes within the cortex/paracortex and vacuolation of the endothelial cells lining the high endothelial venules. After 90 days of recovery, the mean severity score of black particle deposition was similar at 0.25 mg/m^3^ and slightly increased at 5.0 mg/m^3^ (Table 9, Fig. 3 panel f), consistent with continuous drainage of black particles from the lungs after the end of the treatment. In the meantime, the other associated changes disappeared at 0.25 mg/m^3^ and their intensities decreased at 5.0 mg/m^3^.

Cytoplasmic eosinophilic globules (inclusions) in the respiratory and olfactory epithelial cells were observed with increased incidence/severity at 5.0 mg/m^3^ in males and females at both sacrifice time, albeit to lower magnitude after 90 days of recovery indicating partial reversibility (Table 10). Such findings are frequently observed in inhalation studies [33] and are considered to be evidences of irritation [34].

Twenty-four hours post exposure, minimal squamous metaplasia was observed in the larynx in 2 males exposed to 0.25 mg/m^3^ and 4 males and 5 females exposed to 5.0 mg/m^3^. At the end of the 90-day recovery period, minimal squamous metaplasia was still recorded in the larynx of 1 male exposed to 5.0 mg/m^3^ (Table 10). This finding, restricted to the ventral larynx at the base of the epiglottis, is a common reaction to inhaled material and, when of minimal severity, is considered to be a non-adverse adaptive change [35].

No microscopic changes were observed in the respiratory tract of rats exposed to 0.05 mg/m^3^. All other findings observed at 5.0 mg/m^3^ were those commonly seen as spontaneous changes in the rat and bore no relationship to the exposure to Graphistrength^**©**^ C100. Specifically, no histological lesions were observed in pleura (Fig. 3, panel c) and olfactory bulb and no deposit of MWCNT aggregate was observed in the liver, kidneys and bone marrow and other organs of these exposed animals.

### Seminology and spermatid count

Twenty-four hours and 90 days post-exposure, there were no adverse and/or treatment related effects on the sperm counts, motility and morphology (data not shown).

### Micronucleus test

No increase in the frequency of micronucleated polychromatic erythrocytes (PCE) and no signs of medullar toxicity were observed in male and female rats 24 h after 90 days of exposure to 0.05, 0.25 and 5.0 mg/m^3^ of Graphistrength^**©**^ C100 (Table 11). Statistically significant increases in the frequency of PCEs with micronuclei were observed in males and females treated with the positive control CPA.

**Table 11:** Results of the micronucleus assay in the bone marrow of male and female rats 24 h after a 90-day exposure to Graphistrength^©^ C100

Test groups	Concentration / dose level	PCEs with micronuclei (%)	Range^a^	PCE per 2000 erythrocytes
(n = 5/group)	(mg/m^3^)			
**MALE**				
Air control	0	0.340	2 - 17	1099
Graphistrength^©^ C100	0.05	0.440	5 - 11	1073
	0.25	0.280	2 - 10	1094
	5.00	0.210	3 - 8	1081
Positive control^b^	20	0.833*	10 - 26	806
**FEMALE**				
Vehicle	0	0.290	3 - 9	1155
Graphistrength^©^ C100	0.05	0.210	1 - 8	1223
	0.25	0.190	1 - 5	1147
	5.00	0.220	2 - 9	1091
Positive control^b^	20	0.750**	8 - 28	813

*/**: Non-parametric Mann-Whitney test significant <0.05 (*) or <0.01 (**)

^a^per 2000 PCEs per animal

^b^CPA, 20 mg/kg bw

### Comet assay

No increase in the tail intensity (mean of median), in absence and presence of hOGG1, was observed in isolated lung, liver and kidney cells of the male rats 24 h after 90 days of exposure to 0.05, 0.25 and 5.0 mg/m^3^ of Graphistrength^**©**^ C100 (Table 12). Statistically significant increases in percent of DNA in tail were observed in lung, liver and kidney cells of male rats treated with the positive control MMS.

**Table 12:** Results of the comet assay with and without hOGG1 in the lung, kidneys and liver of male rats 24 h after a 90-day exposure to Graphistrength^©^ C100

Test groups (n = 5/group)	hOGG1	Air control	Graphistrength^©^ C100 (mg/m^3^)			Positive control^a^
		0	0.05	0.25	5.0	
**LUNG**						
% of DNA in tail^b^	-	8.8 ± 5.5	3.4 ± 1.5*	8.2 ± 5.1	4.7 ± 3.7	48.7 ± 3.0**
	+	20.2 ± 5.2	16.7 ± 5.7	22.3 ± 11.8	17.9 ± 5.9	79.1 ± 7.9**
Relative ratio of ghost cell^c^	-	-	1.0	1.2	0.8	0.6**
	+	-	2.1**	2.0**	1.3	1.1
**KIDNEYS**						
% of DNA in tail^b^	-	7.4 ± 4.4	7.5 ± 4.2	8.2 ± 4.2	9.7 ± 3.1	72.5 ± 5.8**
	+	22.8 ± 7.4	22.4 ± 7.3	23.4 ± 8.4	24.3 ± 8.0	74.0 ± 5.9**
Relative ratio of ghost cell^c^	-	-	1.6	0.9	1.5	2.8**
	+	-	1.1	1.6*	1.7**	0.6*
**LIVER**						
% of DNA in tail^b^	-	3.8 ± 3.1	3.4 ± 1.6	1.8 ± 1.0	5.5 ± 2.9	71.2 ± 9.1**
	+	11.6 ± 6.4	7.5 ± 1.5	7.7 ± 1.1	9.6 ± 3.0	78.6 ± 3.7**
Relative ratio of ghost cell^c^	-	-	0.2	0.5	2.8**	4.9**
	+	-	0.5*	0.2**	1.1	1.7**

*/**/***: Non-parametric Mann-Whitney test significant <0.05 (*), <0.01 (**)

^a^MMS

^b^Mean of median ± sd

^c^Mean value in treated groups/mean control value

## Discussion

### Inhalation toxicity studies

These 5-day and 90-day inhalation toxicity and genotoxicity studies on Gaphistrength^**©**^ C100 were performed after a careful tuning of the conditions for the generation of a respirable aerosol which respect the physicochemical properties of the MWCNT and allow exposure of all relevant regions of the respiratory tract (MMAD < 3 μm). The procedure that was considered to be most appropriate for this type of agglomerated MWCNT consisted of ball milling and aerosol generation from the sieved material using an aerosol type generator (dust disperser) that minimizes the physical stress to the test material. This approach was subsequently refined by milling the original Gaphistrength^**©**^ C100 under an argon atmosphere to minimize the surface oxidation. An extensive set of physico-chemical investigations was performed and there were generally no relevant modifications between the raw Graphistrength^**©**^ C100 and the aerosol generated from milled and sieved Graphistrength^**©**^ C100. The few changes that occurred were considered to be secondary to the reduction of the particle size and the sieving process. These changes were minor and within acceptable limits considering the procedure used for aerosol generation. In contrast, we observed markedly elevated levels of oxygen surface content in aerosol samples from a previous 5-day inhalation toxicity study with Graphistrength^**©**^ C100 [16] (see the 1). This observation was accompanied by marked surface damages (lace-like appearance) of the MWCNT noticed by TEM which was not apparent by SEM. The principal difference in preparation of the test material consisted in the use of a rotating brush generator which may have scratched the surface of the MWCNT. Surface properties [36, 37] and structural defects [38, 39] being an important factor governing biological effects of MWCNT, the higher levels of the BALF toxicity markers and severity of the lung microscopic changes reported in this study [16] than those observed in our 5-day study are thought to be the consequence of the alteration of the MWCNT by the use of a rotating brush generator.

In both the 5-day and 90-day exposure studies, concentration-related increases of black inclusions (regarded to be MWCNT) were observed in the cytoplasm of infiltrated macrophages, indicating an adequate exposure of the lungs. After 5 days of exposure, the principal findings were limited to the lungs and especially a minimal to slight hypertrophy of the bronchial and bronchiolar epithelial cells and an infiltration of macrophages at 1.25 mg/m^3^. Partial recovery was noted after a 28-day treatment free period. These changes were considered to be a normal physiological response to insoluble particles overload and not adverse.

Twenty-four hours after 90 days of exposure to Graphistrength^**©**^ C100 and also after 90 days of recovery, the signs of systemic effects were limited to an increase in neutrophil counts and a concomitant decrease in lymphocyte counts in blood of rats exposed to 5.0 mg/m^3^ but without change in the total WBC counts. These effects, also observed in a 90-day inhalation study with MWCNT NC 7000 from Nanocyl [40, 41], were most probably secondary to the inflammatory response observed in the lungs of the exposed animals. The microscopic examinations of the heart and aorta, the plasma cholesterol and measurement of the blood pressure after 90 days of exposure did not suggest any cardiovascular changes which could be associated with accelerated progression of atherosclerosis as suggested by Cao *et al.* [17] after 5 weekly i.t. instillations of Graphistrength^**©**^ C100 in wild type and atherosclerosis-prone *ApoE*^−/−^ transgenic mice. As well, the anemic and procoagulant effects reported in Bmal1 (brain and muscle ARNT-like protein-1) knockout (Bmal1^−/−^) mice after 5 weekly oropharyngeal aspirations of Graphistrength^**©**^ C100 [25] were not observed in the rats exposed 90 days by inhalation. The microscopic examination of olfactory bulb and brain sections of the rats exposed to Graphistrength© C100 did not suggest that MWCNT crossed the blood-brain barrier and enter into the olfactory bulb of the brain [42].

After 90 days of exposure to 5.0 mg/m^3^ of Graphistrength^**©**^ C100, a black brown discoloration of the lung and/or greenish foci were seen in most of the rats. This was associated with an increase of around 50 % of the lung weights. The pulmonary reaction to this overload with insoluble particles was revealed by changes in the cytological, biochemical and cytokine parameters of BALF, slight at 0.25 and marked at 5.0 mg/m^3^. All the changes (excepted for TNF-α) observed at 0.25 mg/m^3^ reversed after 90 days of recovery, whereas no clear improvement was observed at 5.0 mg/m^3^. These changes in BALF correlated with a concentration-related deposition of black particle in the lungs, which decreased at 0.05 and 0.25 mg/m^3^ after 90 days of recovery. However, at 5.0 mg/m^3^, the mean severity scores of black particle deposition were overall similar at both time points, indicating a significant overload of the lungs. The increase of alveolar macrophages and the changes in the tracheobronchial lymph nodes at 0.25 and 5.0 mg/m^3^ were consistent with the drainage of the black particles from the lungs [43] and a retention halftime of 375 days estimated in rats exposed for 90 days to 6 mg/m^3^ of MWCNT Baytubes [44]. In animals exposed to 5.0 mg/m^3^, slight inflammatory changes were observed in the lungs at both sacrifice times. The interstitial inflammation mainly around the alveolar ducts at the bronchiole-alveolar junction and the cell hypertrophy/hyperplasia in the terminal and respiratory bronchioles observed just post-exposure were most likely reactive changes to the surrounding inflammatory process. After the 90-day recovery, additional findings were the presence of minimal or slight focal collagen deposition within alveolar septae in a few rats. No microscopic changes were observed in pleura. The minimal squamous metaplasia in the larynx observed 24 h post-exposure to 0.25 and 5.0 mg/m^3^ was fully reversible at 0.25 mg/m^3^ and persisted in one rat at 5.0 mg/m^3^ 90 days post exposure. The presence of epithelial eosinophilic globules in the nasal cavity at 5.0 mg/m^3^ was only partially reversible. All these changes in the respiratory tract by exposure to Graphistrength^**©**^ C100 were qualitatively consistent with those reported in previous 90-day inhalation toxicity studies in rats [40, 41, 44] with two other types of thin and tangled MWCNT produced as large agglomerates like Graphistrength^**©**^ C100. All the pulmonary changes induced by Graphistrength^**©**^ C100 were significantly less severe than those induced in rats exposed for 90 days to 1.0 and 5.0 mg/m^3^ of the long needle-like MWCNT-7 [45], notably the induction of inflammatory and fibrotic effects in the pleura which were not observed in our study.

Pauluhn [43] concluded that a post-exposure period of 6 months (c.a. 0.5 t½) would be suitable to reveal any appreciable reversibility in the lungs after MWCNT exposure. However, fibrotic response was found to develop and persist about one year after an inhalation exposure (4 times/week for 3 weeks) of male C57BL/6 J mice to 5.0 mg/m^3^ of MWCNT-7 [46]. Therefore, the evolution of the inflammatory reaction in the lungs of the rats exposed to Graphistrength^**©**^ C100 for 90 days is still under evaluation over a 1-year recovery period.

The effects of Graphistrength^**©**^ C100 on the lungs of mice were reported by Tabet *et al.* [47] after a single i.t. instillation as micrometric agglomerates suspended in DMEM. The Balb/C mice were then monitored for up to 6 months. BALF analysis showed a dose-dependent increase in total cell count and a significant influx of neutrophils and macrophages only 24 h post-instillation. The MWCNT were internalized in macrophages between 1 day and 1 month after instillation. Histology of the lungs 24 h after instillation showed the presence of widespread micrometric MWCNT agglomerates, which were mainly located in the bronchiolar lumen and alveolar ducts. After one week, clusters of cells surrounding visible MWCNT agglomerates were seen near the terminal bronchioles, the alveolar ducts and alveoli in the lungs. However, no modification in mRNA expression of various genes implicated in oxidative stress (SOD-2 and HO-1), inflammation (CXCL2, TNF-α) and fibrosis (αcollagen-1 and αcollagen-3) was quantified in lung homogenates and no evidence of fibrosis was found 6 months post exposure. Ronzani *et al.* [48] have also assessed the inflammation and airway remodeling in BALF or lung tissue of mice induced by surfactant-dispersed Graphistrength^**©**^ C100, 24 h after a single (6.25 μg/mouse) and 7 days after repeated (1.5, 6.25 and 25 μg/mouse once a week over 3 weeks) intranasal instillation(s). MWCNT distributed all throughout the mouse airways and were observed in alveolar macrophages, epithelial cells, and in infiltrated neutrophils. Mice that received a single administration of MWCNT showed neutrophils infiltrate and greater concentrations of TNF-α, keratinocyte-derived chemokine (KC) and IL-17 in BALF when compared to controls. After repeated Graphistrength^**©**^ C100 administrations, increases in macrophage number, KC and tumor growth factor (TGF)-β1 levels in BALF, and collagen deposition and mucus hyperplasia in lung tissue were observed. Cao *et al.* [17] exposed female wild-type C57BL/6 N Tac mice to total doses of 32 and/or 128 μg Graphistrength^**©**^ C100/mouse administered by i.t. instillation once a week over 5 weeks. Pulmonary inflammation was demonstrated one and/or 28 days after the last exposure by increased influx of neutrophils and higher levels of cytokines (IL1β, IL6, IL12, G-CSF, KC, CCL2, MIP1β, CCL5 and TNF) in BALF, and increased levels of 8-isoprostanes in lung tissue. Even if there are significant methodological differences (rat *vs.* mice, inhalation *vs.* i.t., 90-day exposure *vs.* single or short-term administrations), consistently these studies tend to show the same inflammatory reaction in lungs to insoluble particles.

### Genotoxicity studies

This subchronic inhalation toxicity study gave us the opportunity to evaluate the genotoxic potential of Graphistrength^**©**^ C100 in *in vivo* studies as recommended by the REACH guidance [49] when the set of the available genotoxicity data doesn’t allow a definitive conclusion. The results of *in vitro* studies are conflicting. Graphistrength^**©**^ C100 was negative in a battery of standard *in vitro* genotoxicity assays [50] performed according to the current OECD test guidelines no. 471 [51], 476 [52] and 473 [53] to assess the potential induction of gene mutations in bacteria (Ames test) and mouse lymphoma cells, and chromosomal aberrations in human lymphocytes, respectively. Using nonstandard protocols, Kermanizadeh *et al.* [18, 19] showed that exposure of human hepatoblastoma C3A and HK-2 cells at sub-lethal concentrations of Graphistrength^**©**^ C100 resulted in weak DNA damage in the FPG-modified Comet assay. Conversely, Jackson *et al.* [20] did not observe an increase of the level of DNA strand breaks in the comet assay with FE1-Muta^**©**^ Mouse lung epithelial cell cultures exposed to Graphistrength^**©**^ C100, even if the product was able to generate ROS and the highest tested concentration induced a decrease of the cell proliferation.

In the EU Nanogenotox Joint Action [21], Graphistrength^**©**^ C100 was reported to induce a weak increase (≤2-fold compared to negative control) in the micronucleated binucleated cell (MNBC) frequency in a primary culture of human lymphocytes [22, 23], in adenocarcinomic human alveolar basal epithelial A549 and bronchial epithelial BEAS 2B cell lines [23], but not in the bronchial epithelial 16 HBE and epithelial colorectal adenocarcinoma Caco-2 cell lines [23]. Graphistrength^**©**^ C100 was also tested in the *in vitro* comet assay using BEAS 2B, 16 HBE, A549 and Caco-2 cell lines [23]. In BEAS 2B and Caco-2 cells, Graphistrength^**©**^ C100 was studied using the FpG-modified comet assay. The comet assay was negative for all the cell lines with or without FpG.

The results of the *in vivo* genotoxicity studies published on Graphistrength^**©**^ C100 are scarce and also not conclusive. Cao *et al.* [17] reported a small increase (<2-fold) of the level of DNA strand breaks, without effects on the level of FPG sensitive sites, in lung tissue of dyslipemic C57BL/6 N-Apoe tm1 (*ApoE*^-/-^) mice exposed to Graphistrength^**©**^ C100 by i.t. instillation once a week for 5 weeks at total dose of 128 μg/mouse. This effect was associated with a lung inflammation as demonstrated by an influx of neutrophils in BALF. A 6-fold increase of the mRNA expression of the DNA repair *oxoguanine DNA glycosylase 1* (*Ogg1*) enzyme was also observed in lungs of the same exposed animals. The authors have suggested, according to Risom *et al.* [54, 55], that after repeated exposures to Graphistrength^**©**^ C100, up-regulation of DNA repair counteracts the increased rate of 8-oxodG formation leaving the steady state level of 8-oxodG in DNA unchanged, whereas oxidative DNA damage could be induced after a short-term exposure. However, this hypothesis was not supported by the results of the FPG-modified comet assay performed in the frame of the Nanogenotox Joint Action [24]. Three to six hours after 3 daily i.t. instillations up to 320 μg/rat/day of Graphistrength^**©**^ C100 to Wistar rats, no statistically significant increase in the percentage of tail DNA was noticed in the lung cells with and without FPG enzyme. According to Pant *et al.* [56] a 2- to 10-fold variation of the background levels of DNA damage is not unusual in the comet assay, therefore, it is difficult to ascribe the 2-fold increase reported by Cao *et al.* [17] to Graphistrength^**©**^ C100 exposure. In our study, 24 h after a 90-day inhalation exposure with Graphistrength^**©**^ C100, even in the presence of a clear inflammatory reaction in the lungs, no primary and hOGG1-sensitive oxidative DNA damage was detected by the comet assay, either in the lung cells directly in contact with the MWCNT or systemically in the liver and kidney cells and no micronucleus induction was observed in the bone marrow cells.

The genotoxic effects of MWCNT may result from primary or secondary mechanisms [57]. Primary genotoxicity refers to the elicitation of genetic damage in the absence of inflammation, either by a direct interaction with genomic DNA or associated components that determine its integrity, or indirectly through the enhanced production of ROS by cellular constituents in response to their interaction with particles or through the depletion of antioxidants within the cell which can lead to the induction of oxidative DNA damage [58]. Secondary genotoxicity could be driven by inflammatory cells such as macrophages and polymorphonuclear neutrophilic leukocytes and in situations of chronic inflammation this can lead to persistent oxidative stress and repeated DNA insults [58]. Exposure to some MWCNT has been associated with depletion of antioxidants, increased intracellular production of ROS and pro-inflammatory signaling in cultured cells [59]. In the case of Graphistrength^**©**^ C100 there is no clear evidence of ROS production. An increase was observed in human hepatoblastoma C3A cells [18], but not in human renal [19] or FE1-Muta^**©**^ Mouse lung epithelial [20] cells. After i.t. instillation(s) of Graphistrength^**©**^ C100 in the lung of mice, Tabet *et al.* [47] did not see a modification in mRNA expression of genes implicated in oxidative stress, whereas Cao *et al.* [17] reported an increased expression of *Hmox1*. Even if the ROS formation was not specifically evaluated in our study, the lung inflammation, macrophage phagocytosis, TNF-α secretion in BALF and elevated numbers of neutrophils in blood observed at 5.0 mg/m^3^ are indications of an oxidative stress. The negative comet result in the lung cells exposed to Graphistrength^**©**^ C100 could be related to the ROS scavenger capability of carbon nanotubes [60], which might limit the effects of the oxidative stress, and could also limit the DNA damage. Furthermore, the apparent absence of translocation of Graphistrength^**©**^ C100 from the lungs to the other organs could explain the lack of genotoxic effects in the liver, kidney and bone marrow cells.

Therefore, the lack of *in vivo* genotoxicity of Graphistrength^**©**^ C100 MWCNT indicates a toxicological profile significantly different compared to the genotoxic long needle-like MWCNT-7 [11, 61], classified by IARC [14] as possibly carcinogenic to humans (Group 2B) and to some other genotoxic thin and tangled MWCNT [62–64], even if in these later cases, a role of residual cobalt catalyst, which is a known genotoxin [65], could not be excluded.

## Conclusion

Five-day and 90-day rat inhalation toxicity studies were performed with MWCNT Graphistrength^**©**^ C100. The milling procedure and the dust disperser used as solid aerosol generator produced an aerosol which retained the physico-chemical integrity of the original product in the test aerosols. This contrasts with a previous inhalation study with the same MWCNT product [16], using an aerosol produced with a rotating brush generator in which the structure of the MWCNT was affected. In the present study, principal health findings were limited to the lungs. The infiltration of phagocytizing macrophages is thought to be a trigger and results from the host reaction towards foreign bodies [66]. The inflammation may also deteriorate the alveolar barrier function which increased particle translocation to the draining lymph nodes of the lung [44]. Bronchial and alveolar epithelia were influenced secondarily. During an inhalation study, at a certain time point, a lung burden is reached that exceeds the macrophage clearance capacity and results in overload effects [66]. It seems to be the case at 5.0 mg/m^3^, as Graphistrength^**©**^ C100 deposition persisted in the lung without apparent signs of decrease after a 90-day treatment-free period, whereas at 0.25 mg/m^3^ clear signs of clearance and recovery were observed. Prolonged TNF-α release in BALF was observed at 0.25 and 5.0 mg/m^3^ which was associated only at 5.0 mg/m^3^ with an increased collagen staining like that reported by Pauluhn [44] with MWCNT Baytubes. The lack of genotoxicity in the lung cells and microscopic change in the pleura indicated a lung reaction to Graphistrength^**©**^ C100 exposure quite different than that of the asbestos-like MWCNT-7 and could be related to the absence of internalization of Graphistrength^**©**^ C100 by the alveolar or mesothelial cells as shown by Tabet *et al.* [67] with human epithelial A549 and mesothelial MeT5A cell lines cultures. Overall, these effects are consistent with a normal physiological and not adverse response to the overload of the lung with insoluble particles [44]. Considering the limited and reversible effects on the BALF parameters, the lack of pathological changes in the lungs and the clearance of the Graphistrength^**©**^ C100 observed at 0.25 mg/m^3^, this concentration can be considered as a No-observed Adverse Effect Concentration (NOAEC). In spite of the inflammatory response, neither primary nor oxidative DNA damages were observed locally in lung. The lack of DNA damage or chromosomal aberration remotely in the liver, kidneys and bone marrow was most probably related to the lack of bioavailability *via* a systemic translocation of the MWCNT from the lungs. Therefore, Graphistrength^**©**^ C100 appears of low concern in term of local and systemic genotoxicity and a NOAEC of 0.25 mg/m^3^ (0.28 mg/m^3^ as actual concentration) was established for the repeated-dose toxicity.

## Methods

### General

**Fig. 4:**
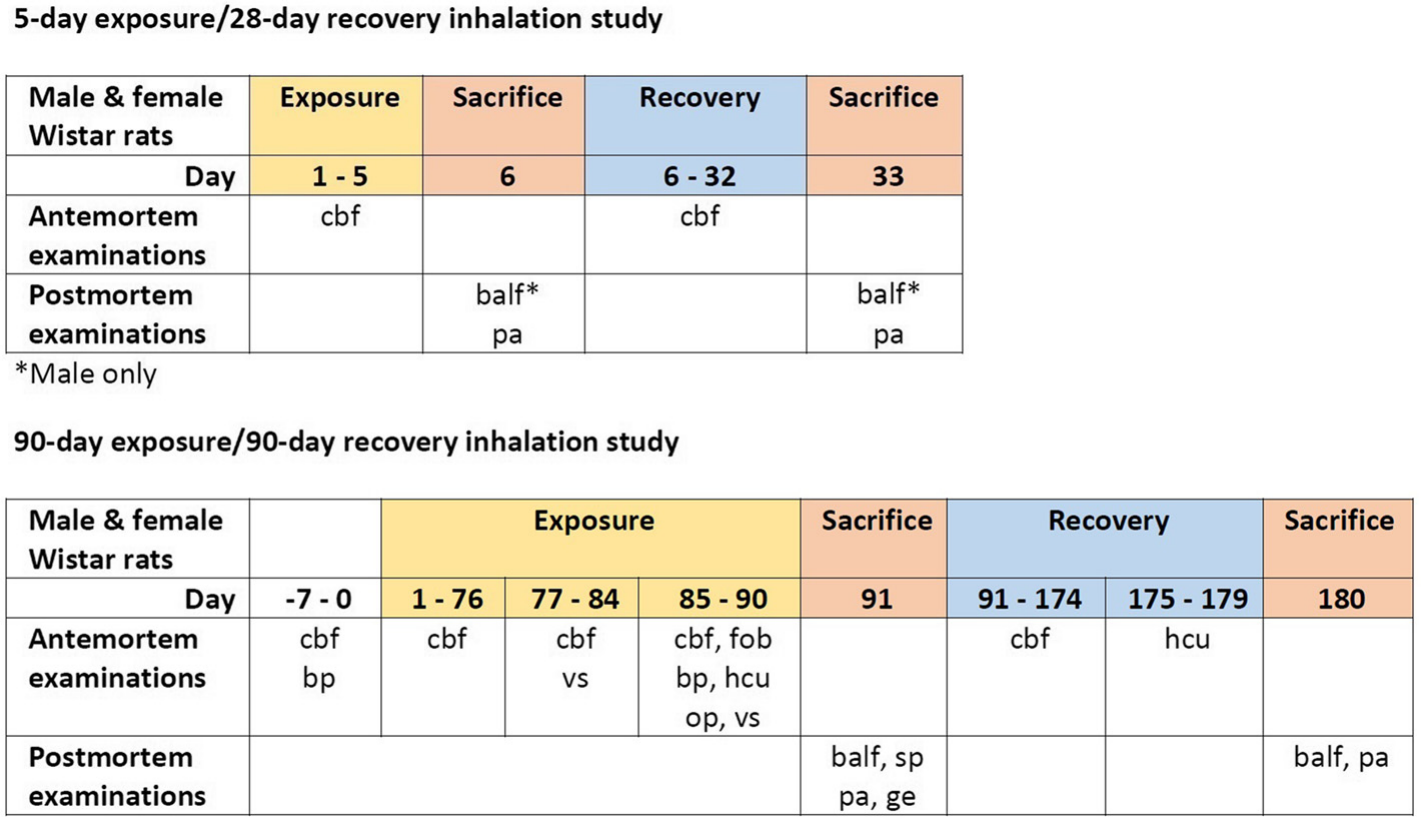
Graphical representation of the design of the 5-day exposure/28-day recovery and 90-day exposure/90-day recovery studies. Abbreviations: balf: bronchoalveolar lavage fluid; bp: blood pressure; cbf: clinical signs, body weight and food consumption; gt: genotoxicity tests (comet and micronucleus assays); hcu: hematology, blood chemistry and urinalysis; fob: functional observation battery; pa: pathology (organ weight, macroscopic and microscopic observations); op: ophthalmology; sp: sperm analysis; vs: vaginal smears

The present studies were conducted according to the OECD Principles of Good Laboratory Practice [68] and the OECD test guidelines no. 413 [29], 474 [69] and 489 [70]. The design of the 90-day inhalation toxicity study was developed taken into account the OECD recommendations [30] for the revision of the tests guidelines applicable to the inhalation toxicity testing of nanomaterials. A graphical representation of the design of the 5- and 90-day studies is presented in Fig. 4. The studies were performed in an AAALAC-accredited laboratory in accordance with the Swiss Animal Protection Law.

### Test materials

**Fig. 5:**
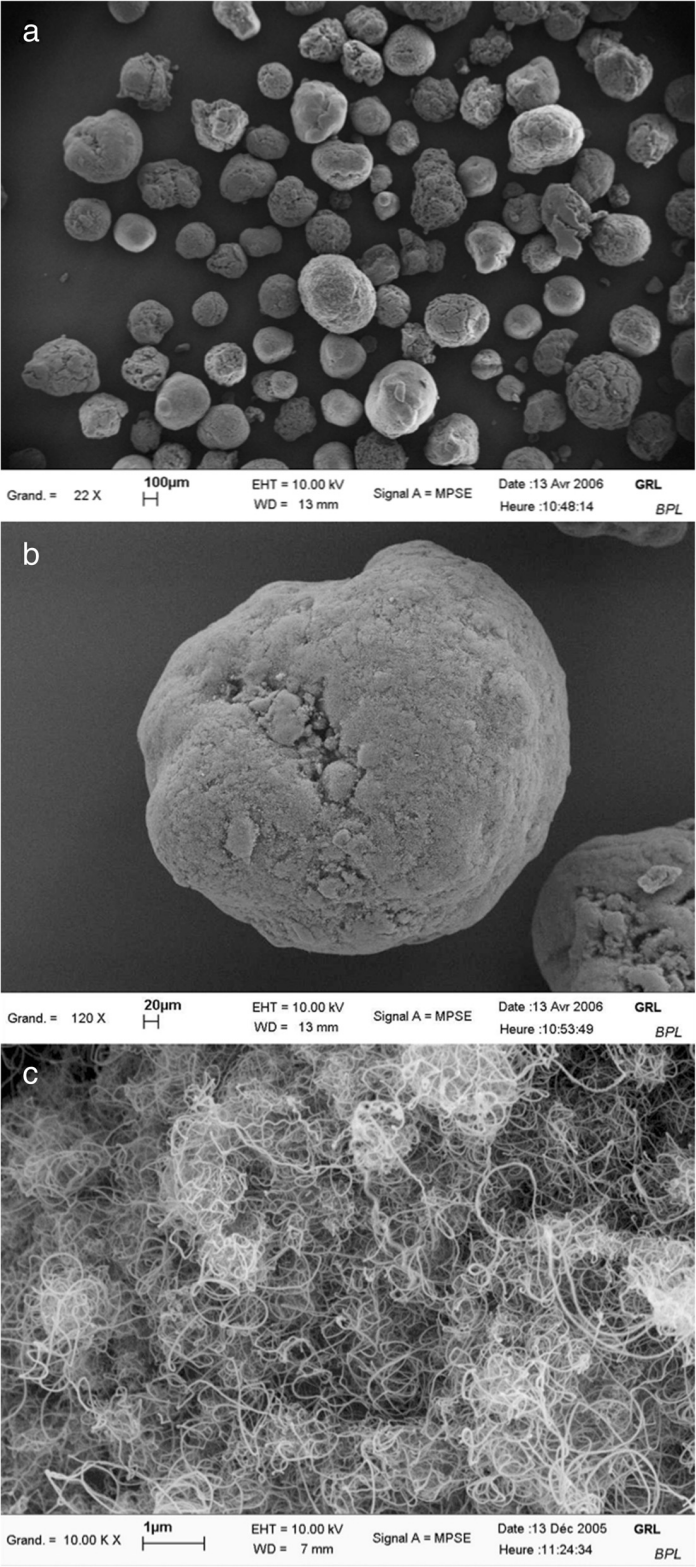
Electron microscopic images of Graphistrength^©^ C100. SEM of the commercial Graphistrength^©^ C100. (**a**) Magnification: 22 fold. (**b**) Magnification: 120 fold. (**c**) Magnification: 10’000 fold

Graphistrength^**©**^ C100, are exclusively produced by Arkema France, Colombes, France. SEM and TEM images (Fig. 5) show that Graphistrength^**©**^ C100 is made of tightly bound agglomerates constituted with entangled MWCNT. These agglomerates can be spherical, ovoid or irregular shaped and have a granulometry centred on 400 μm, with fragments of pellets of less than 15 μm representing a volume of under 0.23 % [27]. The MWCNT of Graphistrength^**©**^ C100 are synthesized at high temperature using a fluidized bed with ethylene as a carbon feedstock and iron oxide (Fe_2_O_3_, ≤5 %, [1309-37-1]) on alumina (Al_2_O_3_, ≤7 %, [1344-28-1]) as catalytic source. Batches nos. 8287 and 110329-018 of Graphistrength^**©**^ C100 were used for the 5-day and the 90-day exposure studies, respectively.

Positive control substances for the genotoxicity assays were selected as recommended by the OECD test guidelines [69, 70]. Cyclophosphamide monohydrate (CPA, batch A0302605, purity 97 %) from Fisher Scientific GmbH for the micronucleus assay and methyl methanesulfonate (MMS, batch MKBL6789V, purity 99.9 %) from Sigma-Aldrich for the comet assay.

### Physico-chemical characterisations

The original Graphistrength^**©**^ C100 batches and samples taken at different steps of our aerosol generation process during the method development (see the additional file 1) were analyzed by SEM (ZEISS LEO 1530 VP Scanning Electron Microscope, LEO Elektronenmikroskopie GmbH, Oberkochen, Germany equipped with an X-rays analyzer Oxford type “Energy 200”) for the morphology of the particles, by TEM (Philips-FEI CM 200 transmission electron microscope, FEI, Hillsboro, Oregon, USA working under electron beam accelerating voltages from 20 kV to 200 kV and with a point-to-point resolution of 0.27 nm) for the walls number, diameters, length size and ends of the nanotubes, by laser method for the particle size, by porosimetry with mercury intrusion for the apparent density, by BET method for specific area (surface to volume ratio was calculated), by calcination for ash content and the elementary organic analysis, by XPS for the chemical surface analysis and by lithium tetraborate fusion method for metal content. Further descriptions of the methods are included in Additional file 1: Table S3.

As reported in the additional file, the effect of the micronisation and aerosolisation methods used for these 5-day and 90-day exposure studies on the integrity and the physico-chemical properties of Graphistrength^**©**^ C100 was carefully examined during the method development. It was also compared to the effect of a brush dust generator as used by Ma-Hock *et al.* [16] during another 5-day inhalation toxicity study with Graphistrength^**©**^ C100 (see the additional file 1).

### Aerosol generation

Graphistrength^**©**^ C100 was ground in a ceramic ball mill for 20 h under air (5-day study) or 12 h under an argon atmosphere to reduce oxidation (90-day study) and the ground test material was sieved before the aerosol generation (see the additional file 1 for details).

The highest aerosol concentrations used for the 5-day and 90-day exposure studies (1.25 and 5.0 mg/m^3^, respectively) were generated from the milled and sieved Graphistrength^**©**^ C100 using a SAG 410 Solid Aerosol Generator (Topas GmbH, Dresden, Germany) connected to a micronizing jet mill (CR Equipement SA, Coppet, Switzerland) and a cyclone and two elutriators (GlasKeller, Basel, Switzerland) thereafter. The effect of the milling duration on the structure (by TEM) and oxidation (by XPS) of the MWCNT was evaluated in a pilot study as reported in the additional file 1. The aerosols generated were then discharged into the exposure chamber through a ^63^Ni charge neutralizer. The generated test aerosols were diluted as necessary with compressed air to achieve the concentrations required for the 5-day and 90-day inhalation exposures. The aerosol concentrations for the low and intermediate groups were achieved by serial dilution with compressed, filtered, dry air of the higher aerosol concentrations (mid and high concentrations, respectively) using a TD190 H and TD190 M air vacuum device (Air-Vac Engineering, Seymour, USA). The aerosol was discharged constantly through the exposure system and exhausted using a tubing/filter system. The exposure system ensured a uniform distribution and provided a constant flow of test material to each exposure tube. The flow of air at each tube was between 0.73 to 1.0 L/min, which was sufficient to minimize re-breathing of the test aerosol as it was more than twice the respiratory minute volume of a rat.

### Animals and husbandry

Healthy male and female Rats, RccHan^**©**^: WIST(SPF) were supplied by Harlan Laboratories, B.V. (5961 NM Horst, The Netherlands). After an acclimatization period of at least 7 days, the animals were 11 and 8 weeks old at the start of 5-day and 90-day exposures, respectively. On the first day of the 5-day and 90-day exposures, the body weights ranged from 291 to 347 g and 243 to 296 g for males and from 176 to 214 g and 135 to 228 g for females, respectively. The rats were randomly allocated by sex to the control and the test groups in groups of maximally five in Makrolon type-4 cages with wire mesh tops and sterilized standard softwood bedding (“Lignocel” J. Rettenmaier & Söhne GmbH & Co. KG, 73494 Rosenberg, Germany) including paper enrichment (Enviro-dri from Lillico, Biotechnology, Surrey, UK). The animal room was air-conditioned with 10-15 air changes/h, a 12 h light-12 h dark cycle, the temperature ranging from 20 to 24 °C with relative humidity ranging from 30 to 70 %. Except during exposure, pelleted standard Harlan Teklad 2914C rodent maintenance diet (Provimi Kliba AG, 4303 Kaiseraugst, Switzerland) and water were provided ad libitum.

### Animal exposure

Groups of 20 male or 10 female rats or 35 male and 35 female rats were exposed nose-only 6 h per day for 5 days or 5 days per week for 90 days, respectively. Target concentrations were 0.05, 0.25 and 1.25 mg/m^3^ air and 0.05, 0.25 and 5.0 mg/m^3^ air of Graphistrength^**©**^ C100 as low, mid and high concentrations for the 5-day and 90-day inhalation exposures, respectively. The choice of the concentrations for the 5-day exposure study was based on published evidence on other MWCNT [40, 44]. Considering the limited effects observed in the 5-day range-finding study, the same low and mid concentrations were used for the 90-day exposure study and the high concentration was increased up to the top concentrations tested in previous 90-day exposure studies [40, 44].

Animals of control groups (0 mg/m^3^) were exposed to compressed air under the same conditions as animals exposed to Graphistrength^**©**^ C100. During nose-only inhalation exposure, rats have a breathing pattern that results in a more realistic internal exposure than a single high dose in i.t. instillation and oropharyngeal aspiration or possible additional oral exposure in whole-body exposure chambers. Inhalation exposure was performed using a flow-past system. The animals were confined separately in restraint tubes which were positioned radially around the flow-past, nose-only exposure chamber as described by Cannon *et al.* [71].

As positive control groups for the genotoxicity assays, 5 males and 5 females were treated once by oral gavage with 20 mg/kg bw cyclophosphamide (CPA) approximately 24 h before tissue sampling for the micronucleus assay, and 5 males were treated by oral gavage with methyl methane sulfonate (MMS) two times with 100 mg/kg bw and then once with 70 mg/kg at approximately 24 h intervals for the comet assay.

### Monitoring and characterization of the test aerosol

The oxygen concentration, relative humidity and temperature in the exposure device were measured continuously during each exposure using a calibrated device. The nominal concentration was determined for the high concentrations by weighing the generator reservoir containing the test material before and after exposure and dividing the used amount by the total air-flow volume. These data were used to monitor the performance of the generation system. During the 5-day and 90-day exposures, gravimetric determinations of the aerosol concentrations were respectively performed once and 4 to 7 times daily for high concentrations and twice and 1 to 3 times daily for the low and mid concentrations using Millipore® durapore filters, type HVLP, loaded in a 47 mm in-line stainless steel filter sampling device. The sampling flow rates were respectively 3 to 4 and 1 L/min per exposure port. For the low concentration of the 90-day exposure study, filters were taken at the inlet pipe with airflow of 3 to 4 L/min. The same device was used to collect aerosol samples for TEM analysis.

For both exposure periods, the cumulative particle size distribution of the test aerosol was determined using Mercer cascade impactors (In-Tox Products, Moriarty, USA). The test aerosol was impacted at each stage (covered with grease) and the particle size distribution of the test material in the generated aerosol was measured by gravimetric analysis of the test material deposited on each stage of the cascade impactor.

During the 5-day exposure, measurements were performed twice for the low and high concentrations and once for the mid concentration using an impactor Model 200 at a sampling rate of 9 L/min in order to collect sufficient material for this short study period.

During the 90-day exposure, measurements were performed at least once per week for the high concentration and five times for the mid concentrations using a standard impactor Model 02-005 at a sampling rate of 1 L/min, matching the air flow rate at the animal port to avoid a possible imbalance of the aerosol flow within the exposure chamber. Accordingly, impactor samples for the mid concentration of the 90-day exposure were collected over several days. No samples were collected for the low concentration of the 90-day exposure as the aerosol concentration was too low to obtain reliable results with an air flow rate of 1 L/min.

The mass median aerodynamic diameter (MMAD) and the geometric standard deviation (GSD) were calculated on the basis of the results from the impactors, using Microsoft Excel® software (Microsoft Corporation, USA). In addition, during the 90-day inhalation exposure, the aerosol was analyzed once per week with a Wide Range Particle Spectrometer^**©**^ (WRPS, Model 1000XP, MSP Corporation, Shoreview, USA) in the size range of 5 nm to 10 μm to determine the count median aerodynamic diameter (CMAD). The sampling airflow rate was approximately 1 L/min.

### Ante mortem observations

Cage-side clinical observations were recorded twice daily before and after exposures. During the 90-day study, a careful clinical examination of each animal was performed once weekly in a standard arena. Food consumptions and body weights were recorded twice during the 5-day exposure and weekly during the 28-day recovery and the 90-day exposure and 90-day recovery periods.

Functional observation battery, locomotor activity, grip strength, landing foot splay, body temperature and ophthalmoscopic investigations were recorded during the last week of the 90-day exposure period. In animals fasted in metabolism cages for approximately 18 h, hematology, blood chemistry and urinalysis (parameters as per OECD test guideline no. 413 [29] and listed in Tables 2 and 3) were performed during the last week of the 90-day exposure. Hematology and blood chemistry were repeated during the last week of the 90-day recovery period. Tail cuff blood pressure was measured during acclimatization and during the last week of the 90-day exposure period. Vaginal smears for estrous cycle evaluation were taken for 14 days from all females during treatment weeks 11 and 12.

### Post mortem observations

Twenty-four hours and 28 days after the 5-day exposure, 5 rats/sex/group were anaesthetized by i.p. injection of pentobarbitone and killed by exsanguination for histological examinations and another 5 males/group served for BALF investigations on both occasions. Twenty-four hours and 90 days after the 90-day exposure, 10 rats/sex/groups were sacrificed for organ weights, and macroscopic, histological and BALF examinations.

#### Bronchoalveolar lavage fluid analysis

For broncho-alveolar lavage sampling, the complete lungs (5-day study) or the left lobes of the lungs (90-day study) were washed six times with physiological saline (4.0 ml for the complete lungs or 1 x 2.5 mL + 5 x 1.5 mL for the left lobes) at room temperature by slow instillation and withdrawal of fluid. The lavage fluid of animals from the positive control group for the comet assay was discarded. After washing, the left lung lobes were preserved in mincing solution at 5 ± 3 °C for the comet assay. The lavage fluids from the first (5-day study) or the first two lavages (90-day study) were collected in a centrifugation tube on ice. The recovered volume from this wash was recorded. Approximately 0.75 or 1 mL of the fluid was placed into a second tube containing 37.5 or 50 μL of 20 % BSA in PBS, respectively. Both tubes were centrifuged at approximately 300 g for 10 min between 2 and 8 ° C. Thereafter, the supernatant (without BSA) was taken in one tube and immediately set on ice and stored at 2-8 ° C until analysis. The first aliquot (without BSA) was used for the determination of the enzymatic activity of lactate dehydrogenase (LDH), alkaline phosphatase (ALP), and γ-glutamyltransferase (GGT) as well as for the determination of total protein and/or phospholipids (storage at 2-8 ° C until analysis). The second aliquot from the 90-day exposed and 90-day recovery animals was split into three tubes, two of them containing approximately 0.1 mL each, the third one containing 0.3 mL and were analyzed for contents of natural immunity mediator cytokines (TNF-α, IL-1α and IL-1β) and adaptive immunity mediator cytokines (IL-5). IL-5, IL-1-β and TNF-α were analyzed using the Proinflammatory Panel 1 (rat) Kit V-Plex^TM^ from MSD. IL-α was analyzed using the MSD® Multi-Spot Assay System, Rat Demonstration 7-Plex Ultra-sensitive Kit.

The lavage fluid from the four further lavages was pooled separately for each animal in a centrifugation tube, and centrifuged at approximately 300 g for 10 min between 2 and 8 °C. The supernatant was discarded. The cell pellets were each suspended with physiological saline and pooled with the suspended cell pellet from the first two lavages. The total volume was gently shaken for mixing. Aliquots of the cell suspension were taken for total cell count, cell viability and differential cell counting. A total cell count was performed by diluting an aliquot of the cell suspension in Türk’s stain (Diagnostik Merck No. 9277, Merck AG, Darmstadt, Germany). An aliquot of this mixture was introduced into a hemocytometer chamber, and the cells were counted (at least 1 mm^3^). A differential cell count was performed. According to the results of the total cell count, an aliquot of cell suspension was diluted with physiological saline solution to give an end concentration of at least 10^6^ cells/ml. From this suspension a smear was prepared using a Cytocentrifuge (Shandon, Instrument Gesellschaft AG, Switzerland) and stained with Diff-Quick (Baxter Dade, Switzerland). At least 500 cells per smear were counted by light microscopy. The number of each cell type (macrophages, neutrophils, lymphocytes, eosinophils, epithelial cells, and other cells) was counted.

#### Macroscopic and organ weight examinations at necropsy

All animals were weighed at necropsy and descriptions of all macroscopic abnormalities were recorded. The weights of adrenal glands, heart including auricles, kidneys, liver, lungs, and spleen were determined in all animals exposed for 5 days and 90 days (including recovery animals). In addition, weights of brain, epididymis, ovaries, testes, thymus and uterus were recorded in all animals exposed for 90 days (including recovery animals). The organ to terminal body weight ratios was calculated.

#### Histopathology

Almost all organs were collected from all rats exposed for 5-day (including recovery animals). All gross lesions, all organs as per OECD test guideline no. 413 [29], olfactory bulb and pleura were collected from all rats exposed for 90 days (including recovery animals). Lung lobes were instilled *via* trachea with neutral phosphate buffered 4 % formaldehyde solution at approximately 30 cm H_2_O pressure. The organs were fixed in formalin (excepted testes fixed in modified Davidson’s solution), processed, embedded and cut at an approximate thickness of 2 - 4 μm and stained with hematoxylin and eosin.

For the 5-day exposure study, all gross lesions, heart including auricles, kidneys, larynx, liver, spleen, mediastinal lymph nodes, nasal cavity, nasopharyngeal duct and pharynx, spleen and trachea (adjacent to larynx and carina and bifurcation) of the main and recovery rats from the control and 1.25 mg/m^3^ groups were examined microscopically. Lungs from all main and recovery rats from all groups were examined.

For the 90-day exposure study, the microscopic examination of the respiratory tract and associated lymph nodes was performed on all control and exposed animals at both sacrifice periods (except the nasal cavity and larynx of the recovery rats exposed to 0.05 mg/m^3^). The other organs, as per OECD test guideline no. 413 [29], were examined for the control and 5.0 mg/m^3^ groups rats sacrificed 24 h post-exposure (this microscopic examination was not performed on the 90-day recovery rats as no microscopic changes was observed in the 24-h sacrificed rats).

A part of left lobe of lungs (also used for BALF), a caudal part of kidney and the middle lobe of liver of male rats exposed for 90 days were preserved for the comet assay.

#### Semiology and spermatid count

Sperm motility, spermatid count in testis and sperm count in cauda epididymis were performed on all males exposed for 90 days and sacrificed 24 h and 90 days post exposure (expect the sperm count in recovery males exposed to 0.05 and 0.25 mg/m^3^). Sperm morphology was analyzed on all males at the 24-h post exposure sacrifice and on control and 5.0 mg/m^3^ exposed males after the 90-day recovery period.

#### Micronucleus test

The 5 last male and female rats (in the numbering order) out of 10 of sacrificed 24 h after the 90-day exposure were used for the micronucleus assay. The femora were removed, the epiphyses cut off and the marrow was flushed out with fetal calf serum, using a syringe (3 mL per femur). The nucleated cells were separated from the erythrocytes using the method of Romagna [72]. Briefly, the cell suspensions were passed through a column consisting of α-Cellulose (Sigma) and Cellulose (Sigmacell type 50) (1:1 mixture). The columns were then washed with Hank΄s buffered saline. The cell suspensions were centrifuged at 1500 rpm (390 × g) for 10 min and the supernatant was discarded. The pellet was suspended in a small drop of fetal calf serum and spread on slides. The smears were air-dried, fixed in methanol. The slides were stained with May-Grünwald/Giemsa. For staining, the slides were first incubated one minute in a 5 % May-Grünwald solution followed by one minute in a 1:1 mixture of May-Grünwald/phosphate buffer (pH 7.4). After 20 min in 14 % Giemsa solution the slides were washed twice in phosphate buffer and 10 s in deionized water. Cover slips were mounted with EUKITT (Kindler, 79110 Freiburg, Germany). At least one slide was made from each bone marrow sample. The slides were coded using a computer generated coding list. Evaluation of the slides was performed using NIKON microscopes with 100x oil immersion objectives. Six thousand polychromatic erythrocytes (PCE) were analyzed per animal for micronuclei.

The test is considered to be positive if there is either a dose-related increase in the number of micronucleated polychromatic erythrocytes or a statistically significant positive response for at least one of the test points. In the absence of a dose-related increase in the number of micronucleated polychromatic erythrocytes or a statistically significant positive response at any of the test points the substance is considered non-genotoxic in this system. To describe a cytotoxic effect the ratio between polychromatic and normochromatic erythrocytes was determined in the same sample and expressed in polychromatic erythrocytes per 2000 erythrocytes.

#### Comet assay

As suggested by the OECD test guideline no. 489 [70], when there was no difference in toxicity between males and females, the comet assay could be performed in either sex. The 5 first male rats (in the numbering order) out of 10 sacrificed 24 h after the 90-day exposure were assigned for the comet assay. Three to 6 h after the last treatment for positive control group or 22 to 26 h after the last exposure for air control and Graphistrength^**©**^ C100 exposed groups, the lung (left lobe used for BALF), the kidney (caudal part) and the liver (middle lobe) were collected from each animal and maintained in cold mincing buffer (Mg^2+^ and Ca^2+^ free Hanks’ Balanced Salt Solution (Gibco, CA, USA) with 20 mM Na EDTA (EDTA) (Sigma, USA) and 10 % (v/v) dimethylsulfoxide (DMSO) (Sigma)). Immediately after dissection, the organs were minced using fine scissors in cold mincing buffer. The cell suspension was stored on ice for 15-30 s to allow large clumps to settle, and the supernatant was used to prepare comet slides. An aliquot of single cell suspension (3 x 10^4^ cells) was mixed per 75 μL of 0.5 % low melting agarose (Invitrogen, USA) and spread on comet assay slides (Travigen, USA). The slides were immersed in cold lysis solution (2.5 M NaCl, 100 mM Na_2_EDTA, 10 mM Tris-base, 10 % DMSO, 1 % Triton-X (pH 10)) overnight. For the hOGG1-modified comet assay, following lysis as described, slides were washed two times for 5 min with the hOGG1 incubation buffer (40 mM Hepes, 100 mM KCl, 0.5 mM EDTA and 0.2 mg/mL BSA) at room temperature and then incubated for 10 min at 37 °C with 0.12 x 10^-3^ U/slide of hOGG1 (Biolabs) in the hOGG1 incubation buffer. After this incubation period, slides were placed in electrophoresis solution (0.3 M NaOH, 1 mM EDTA (pH > 13)) for 20 min to allow for DNA unwinding. Subsequently, electrophoresis was conducted at 25 V, 300 mA for 20 min. The slides were immersed in a neutralization solution (0.4 M Tris-base, pH 7.5) for at least 5 min and dehydrated with absolute ethanol to fix. The cells were stained with propidium iodide (20 μg/mL) (Invitrogen). All slides were independently coded before the microscopic analysis. The comet was observed *via* fluorescence microscope (Leica Microsystems SAS - DM 2000, Heerbrugg, Switzerland) at magnification of x200 and analyzed by COMET ASSAY IV software (Perceptive Instruments, UK). For each sample (animal/tissue), fifty comets per slide were analyzed, with 3 slides scored per sample.

A positive response is defined as a statistically significant change in the % tail DNA in at least one dose group in comparison with the vehicle control value. The positive control should produce a statistically significant increase. Cytotoxicity was evaluated by the enumeration of hedgehogs. Indeed, important fragmentation of the DNA can be induced not only by genotoxicity but also during the process of cell death, *i.e.* apoptosis and necrosis.

### Statistical analysis

The food consumption, blood pressure, grip strength, landing foot play, body temperature, body weight, food consumption, blood clinical laboratory and BALF parameters, organ weights, and sperm parameters were analyzed for statistical significance by the Dunnett-test (many to one *t*-test) based on a pooled variance estimate, if the variables can be assumed to follow a normal distribution for the comparison of the treated groups and the control groups for each sex. The Steel-test (many-one rank test) was applied for the locomotor activity, urinalysis, and BALF biochemical parameters instead of the Dunnett-test when the data cannot be assumed to follow a normal distribution. Fisher’s exact test was applied to the ophthalmoscopy and macroscopic findings. Nonparametric Mann-Whitney test was applied to the micronucleus, comet and to cytokines test results as data did not follow a normal distribution.

## Additional file

Additional file 1: Figure S1. Electron microscopic images of Graphistrength^©^ C100 aerosols generated by the rotating brush generator [16]. (A) TEM of the original material batch no. 6097, Magnification: 135’000 fold. (B) and (C) TEM of aerosol samples. Magnification: 135’000 fold. (D) SEM of aerosol sample, Magnification: 25’000 fold. **Figure S2:** Scanning (SEM) and transmission (TEM) electron microscopic images of Graphistrength^©^ C100 aerosols generated by the dust disperser and collected directly into TEM grids. Millipore® durapore filter, Type HVLP as used in the 5-day inhalation study. (A) SEM, Magnification: 2’000 fold. (B) SEM, Magnification: 29’500 fold. (C) TEM, Magnification: 350’000 fold. **Figure S3:** Particle size distribution after different milling durations of Graphistrength^©^ C100. **Figure S4:** Scanning (SEM) and transmission (TEM) microscopic images of Graphistrength^©^ C100 using the refined aerosol generation procedure used during the 13-week exposure study. (A) TEM of original Graphistrength^©^ C100 batch no. 110329-018, 350’000 fold. (B) TEM of milled Graphistrength^©^ C100 under argon for 12 h and sieved (63 μm), 350’000 fold. (C) TEM of aerosol sample, 350’000 fold. (D) SEM of aerosol sample, 50’000 fold. **Figure S5:** BALF parameters of male rats 24 h (A) and 4 weeks (B) after a 5-day exposure to Graphistrength^©^ C100. Changes are shown as x-fold differences compared to controls using a logarithmic scaling. Abbreviations: ALP: alkaline phosphatase, GGT: γ-Glutamyltransferase, LDH: lactate dehydrogenase. **Figure S6:** Microscopic appearance of lungs around terminal bronchiole in rats after a 5-day exposure to Graphistrength^©^ C100 (A) Control. (B) Graphistrength^©^ C100, 1.30 mg/m^3^, after a 5-day inhalation exposure: Terminal bronchiolar epithelium showing hypertrophic appearance (asterisks). No inflammatory and granulomatous lesions are found. (C) Graphistrength^©^ C100, 1.30 mg/m^3^, after a 5-day inhalation exposure: Blackish particle laden alveolar macrophages (arrows). (D) Graphistrength^©^ C100, 1.30 mg/m^3^, after a 4-week recovery period: Histologic appearance of the bronchiolar epithelium recovered partially. Original lens magnification: (A), (B) and (D) Magnification: 10 fold; (C) Magnification: 40 fold. Table S1: Oxygen surface content by XPS in Graphistrength^©^ C100 aerosols from the rotating brush dust generator [16]. **Table S2:** Effect by XPS of the milling duration and milling atmosphere (air or argon) on the oxygen surface content in Graphistrength^©^ C100. **Table S3:** Physico-chemical characterization of Graphistrength^©^ C100 before and after aerosol generation. **Table S4:** Target and achieved aerosol concentrations and particles size of Graphistrength^©^ C100. Temperature, relative humidity and oxygen concentration measured over the 5-day exposure study. **Table S5:** Cellular analysis of BALF of male rats 24 h and 4 weeks after a 5-day exposure to Graphistrength^©^ C100. **Table S6:** Biochemical analysis of BALF of male rats 24 h and 4 weeks after a 5-day exposure to Graphistrength^©^ C100. **Table S7:** Main microscopic findings in the lungs of male and female rats 24 h and 4 weeks after a 5-day exposure to Graphistrength^©^ C100.

## Abbreviations

AAALAC: Association for Assessment and Accreditation of Laboratory Animal Care
ALP: Alkaline phosphatase
BALF: Broncho-alveolar lavage fluid
BET: Brunauer, Emmett and Teller (method)
BSA: Bovine serum albumin
CCL2: Chemokine ligand 2
CCL5: Chemokine (C-C motif) ligand 5
CMAD: Count median aerodynamic diameter
CPA: Cyclophosphamide
CXCL2: Macrophage inflammatory protein-2
DMEM: Dulbecco’s modified Eagle medium
DNA: Deoxyribonucleic acid
ESCA: Electron spectroscopy for chemical analysis
FPG: Formamidopyrimidine DNA glycosylase
GGT: γ-glutamyltransferase
GPX-1: Glutathione peroxidase-1
G-CSF: Granulocyte colony-stimulating factor
GSD: Geometric standard deviation
HCD: Historical control data
hOGG1: Human 8-hydroxyguanine DNA-glycosylase
HO-1: Heme oxygenase-1
IARC: International Agency for Research on Cancer
IL: Interleukin
KC: Keratinocyte-derived chemokine
LDH: Lactate dehydrogenase
MIP1β: Macrophage inflammatory protein 1β
MMAD: Mass median aerodynamic diameter
MMS: Methyl methanesulfonate
MWCNT: Multiwall carbon nanotubes
OECD: Organization for Economic Cooperation and Development
PBS: Phosphate buffered saline
PCE: Polychromatic erythrocytes
PMN: Polymorphonuclear neutrophils
ROS: Reactive oxygen species
SEM: Scanning electron microscopy
SOD: Superoxide dismutase
TEM: Transmission electron microscopy
TNF-α: Tumor necrosis factor alpha
WBC: White blood cells
WPRS: Wide Range Particle Spectrometer^**©**^
XPS: X-ray photoelectron spectroscopy
