# Supplementary material for: Lung inflammation and lack of genotoxicity in the comet and micronucleus assays of industrial multiwalled carbon nanotubes Graphistrength© C100 after a 90-day nose-only inhalation exposure of rats
Source: Part Fibre Toxicol. 2015 Jul 10;12:21. doi: 10.1186/s12989-015-0096-2 (PMC4496819; doi:10.1186/s12989-015-0096-2)
Supplement: Additional file 1: Figure S1. — Electron microscopic images of Graphistrength© C100 aerosols generated by the rotating brush generator [16]. (A) TEM of the original material batch no. 6097, Magnification: 135’000 fold. (B) and (C) TEM of aerosol samples. Magnification: 135’000 fold. (D) SEM of aerosol sample, Magnification: 25’000 fold. Figure S2: Scanning (SEM) and transmission (TEM) electron microscopic images of Graphistrength© C100 aerosols generated by the dust disperser and collected directly into TEM grids. Millipore® durapore filter, Type HVLP as used in the 5-day inhalation study. (A) SEM, Magnification: 2’000 fold. (B) SEM, Magnification: 29’500 fold. (C) TEM, Magnification: 350’000 fold. Figure S3: Particle size distribution after different milling durations of Graphistrength© C100. Figure S4: Scanning (SEM) and transmission (TEM) microscopic images of Graphistrength© C100 using the refined aerosol generation procedure used during the 13-week exposure study. (A) TEM of original Graphistrength© C100 batch no. 110329-018, 350’000 fold. (B) TEM of milled Graphistrength© C100 under argon for 12 h and sieved (63 μm), 350’000 fold. (C) TEM of aerosol sample, 350’000 fold. (D) SEM of aerosol sample, 50’000 fold. Figure S5: BALF parameters of male rats 24 h (A) and 4 weeks (B) after a 5-day exposure to Graphistrength© C100. Changes are shown as x-fold differences compared to controls using a logarithmic scaling. Abbreviations: ALP: alkaline phosphatase, GGT: γ-Glutamyltransferase, LDH: lactate dehydrogenase. Figure S6: Microscopic appearance of lungs around terminal bronchiole in rats after a 5-day exposure to Graphistrength© C100 (A) Control. (B) Graphistrength© C100, 1.30 mg/m3, after a 5-day inhalation exposure: Terminal bronchiolar epithelium showing hypertrophic appearance (asterisks). No inflammatory and granulomatous lesions are found. (C) Graphistrength© C100, 1.30 mg/m3, after a 5-day inhalation exposure: Blackish particle laden alveolar macrophages (arrows). (D) Graphistrength© C100, [file 12989_2015_96_MOESM1_ESM.docx]

**Method development for Aerosol Generation**

Methods have been developed to generate respirable aerosols from MWCNT produced as large agglomerates like Graphistrength^©^ C100. A rotating brush dust generator was used by Ma-Hock *et al.* in 5-day and 13-week inhalation exposure studies with Graphistrength^©^ C100 [16] and Nanocyl’s MWCNT [40], respectively. Briefly, MWCNT powder is loaded into a cylindrical feed stock reservoir and is fed onto a rotating brush by means of a piston. The rotating brush picks up the MWCNT from the surface of the compacted powder charge. The aerosol is dispersed and is carried away from the dispersion head with compressed filtered air and diluted with conditioned air and passed via a cyclone into the exposure chamber. We have assessed the effects of the rotating brush dust generator on the structure and physicochemical properties of Graphistrength^©^ C100 MWCNT (batch no. 6097) by X-ray photoelectron spectroscopy (XPS) and transmission electron microscopy (TEM). Some aerosol samples were collected at the cyclone and exhaust of the inhalation chamber, under the same conditions as in the 5-day inhalation study [16], and were generously provided by the principal investigator of this study. XPS analysis of the samples showed a significant increase (at least 8-fold) in the oxygen surface content as shown in Table s1 compared to the original Graphistrength^©^ C100. In addition, the surface structure of the MWCNT showed significant physical alterations with a lace-like appearance as can be seen in Figure s1.

Since the rotating brush dust generator seemed inadequate to generate a respirable aerosol without alteration of the Graphistrength^©^ C100 MWCNT structure, an alternative method was developed similar to a procedure published by Pauluhn [44]. During the initial method development for the 5-day range-finding study, the test material (batch nos. 8287) was milled in a ball mill (Morgan Technical Ceramics, Waldkraiburg, Germany) for various durations between 6 and 96 hours to increase the dustiness. The volume of the ball mill was 500 ml and 50 ceramic balls were used (20 of 10 mm, 15 of 20 mm and 15 of 30 mm). The oxygen content at the surface of the test material before and after milling was determined by XPS.

The results in Table s2 demonstrate that no surface oxidation occurred up to 24 hours and increased oxygen content was observed only after 48 hours of milling. Accordingly, the duration for milling in further experiments was set at a maximum of 20 hours. The efficiency of the milling was investigated by sieving through different sieve sizes using an AS200 Digit sieve shaker (Retsch GmbH, Haan, Germany). All the 20-hour ball milled material passed the sieve size of 63 µm while only 22% of the bulk material did so. The effect of sieving on the oxygen surface content was determined by XPS analysis on the different sieved fractions (< 63 to > 200 µm) obtained from the bulk material and the 96-hour milled product. The oxygen surface content of the fractions from the bulk test material was comparable to the non-sieved product and there was no difference between the 63-100 µm and < 63 µm fractions of the milled product (2.06 vs. 2.28%). The fraction below 63 µm was used for the atmosphere generation in all the inhalation toxicity studies.

During a technical trial, the aerosol was collected on filters from open ports of the exposure chamber and investigated by TEM and scanning electron microscopy (SEM). During this trial, the aerosol concentration was 18 mg/m^3^ air and the determination of the particle size distribution resulted in mass median aerodynamic diameters (MMAD) of 2.93 μm and 3.55 μm. There was no indication that the integrity of the MWCNT was affected as shown in Figure s2 and the external (11.9 ± 3.3 nm) and internal (4.3 ± 1.7 nm) diameters and the length (mean 883 nm; range 90 nm - 4550 nm) were not significantly changed compared to the original Graphistrength^©^ C100 before micronisation (see Table s3 for batch no. 8287). Therefore this test material preparation was considered suitable for the 5-day inhalation toxicity study.

After completion of the 5-day study, the milling procedure was done in an argon atmosphere in order to further reduce the risk of oxidation. The test material was transferred into the ball mill container inside a barrel flushed with argon at a level of less than 1% oxygen. Thereafter, the whole ball mill was placed inside a containment filled with argon and kept at an oxygen level of generally below 2%. Physical characterization of samples milled in an argon atmosphere is detailed in Table s3. The analysis of the oxygen surface content by XPS showed only an increase by 0.3% compared to 1.0% under standard atmosphere conditions (Table s1) after a 96-hour milling. For batch no. 110329-18 used in the 13-week exposure study, the iron and aluminum contents were comparable between the raw material and milled sample (Table s3). Further experiments on milling were performed and the particle size distribution of the test material was determined after different durations up to 24 hours as detailed in Figure s3. There were significant differences in the size of the particles between the bulk material (D_10_ = 223 µm, D_50_ = 418 µm) and the milled product (D_10_ = 8.20-12.0 μm, D_50_ = 25.2-30.6 μm) irrespective of the milling duration and there was a small increase of particles smaller than 0.1 µm beyond 6 hours of milling. Accordingly, a 12-hour milling duration using argon as a protection atmosphere was chosen for the 13-week exposure study. The results of the physico-chemical characterization of Graphistrength^©^ C100 before and after a 12-hour milling under argon and after aerosol generation (samples collected at the exhaust of the elutriator just before the inhalation chamber) are presented in Table s3 and SEM and TEM images in Figure s4. These analyses comprised apparent density, elementary organic analysis, specific area, metal content by ICP, chemical surface analysis by XPS, external diameters, wall numbers, nanotube length, and surface to volume ratio. Minor changes were noted between the starting material and the ball milled or aerosol samples and can be divided in two groups. For apparent density, surface to volume ratio, and MWCNT length, the changes observed are inherent to the aerosol form. When modifying particle size of the nanomaterial, these characteristics are necessarily changed. The original, ball milled and micronised Graphistrength C100 were in the form of balls of entangled MWCNT. These balls had rather a spherical shape and their size depended on the sample. They were around 400 µm diameter for the reference sample whereas they were much smaller for milled and micronised samples (Figure s4 panel D). For these last samples, MWCNT balls are rather agglomerated. These differences are consistent with an aerosol for which agglomerate size is necessarily smaller. There was no apparent alteration by TEM of the MWCNT structure between the original, milled and aerosolized Graphistrength^©^ C100 (Figure s4, panels A, B and C). On the other hand, a very low (<50 ppm) metal pollution with chromium and nickel was found and was attributed to the sieving procedure where stainless steel materials were used.

# **FIGURES**

## Figure s1 – Electron microscopic images of Graphistrength^©^ C100 aerosols generated by the rotating brush generator [16]

**
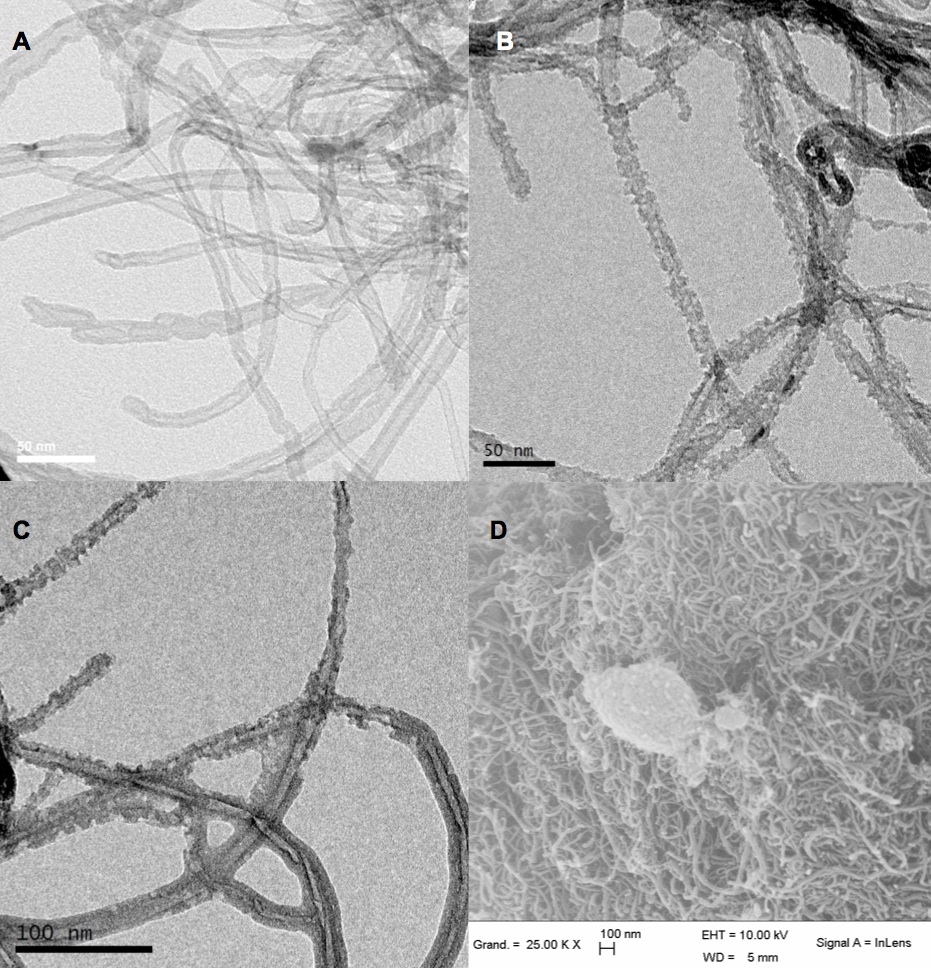
**

**(A)** TEM of the original material batch no. 6097, Magnification: 135’000 fold. **(B)** and **(C)** TEM of aerosol samples. Magnification: 135’000 fold. **(D)** SEM of aerosol sample, Magnification: 25’000 fold.

**Figure s2 – Scanning (SEM) and transmission (TEM) electron microscopic images of Graphistrength**^©^ **C100 aerosols generated by the dust disperser and collected directly into TEM grids**

Millipore^®^ durapore filter, Type HVLP as used in the 5-day inhalation study. **(A)** SEM, Magnification: 2’000 fold. **(B)** SEM, Magnification: 29’500 fold. **(C)** TEM, Magnification: 350’000 fold.


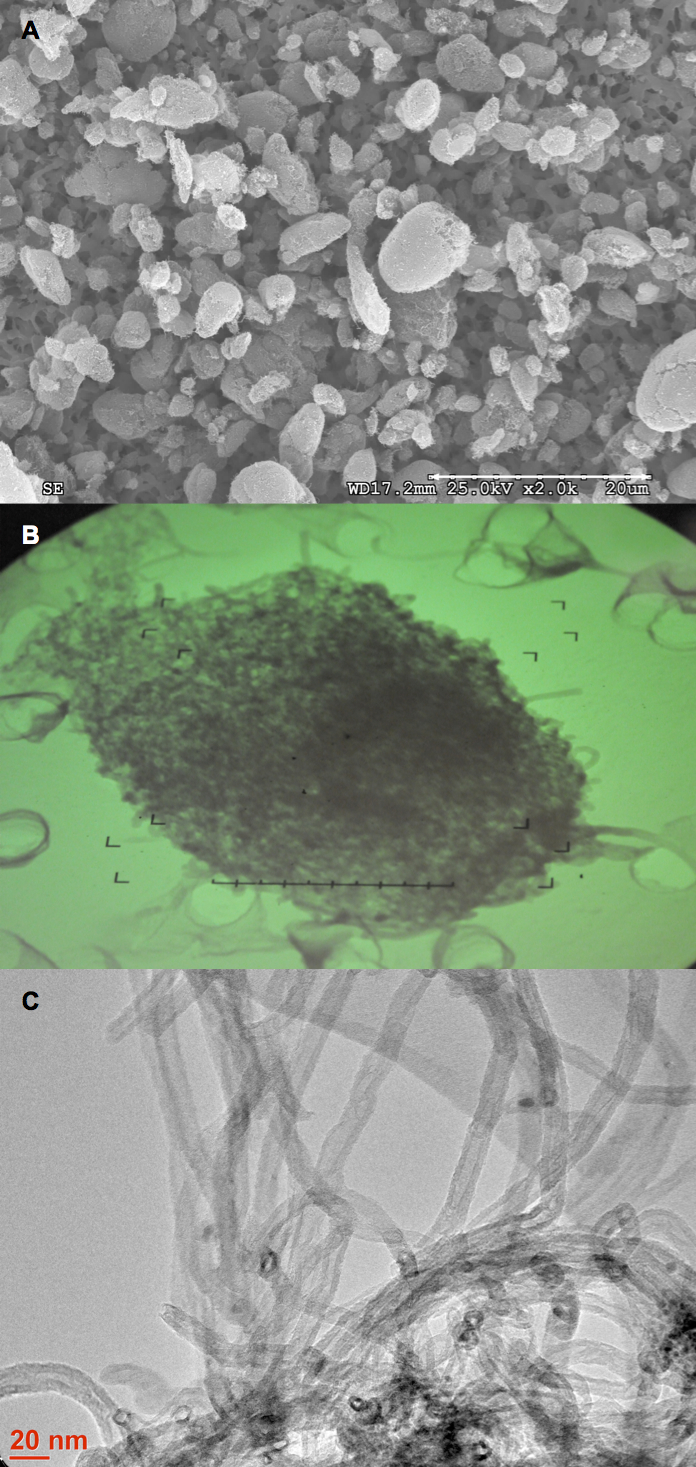


## Figure s3 – Particle size distribution after different milling durations of Graphistrength^©^ C100


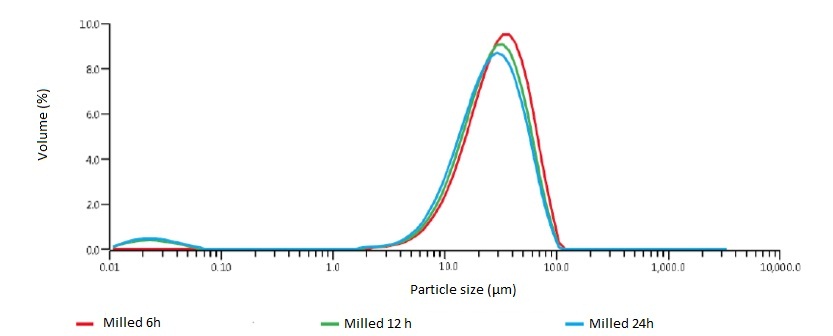


## Figure s4 – Scanning (SEM) and transmission (TEM) microscopic images of Graphistrength^©^ C100 using the refined aerosol generation procedure used during the 13-week exposure study

**(A)** TEM of original Graphistrength^©^ C100 batch no. 110329-018, 350’000 fold. **(B)** TEM of milled Graphistrength^©^ C100 under argon for 12 h and sieved (63 µm), 350’000 fold. **(C)** TEM of aerosol sample, 350’000 fold. **(D)** SEM of aerosol sample, 50’000 fold.


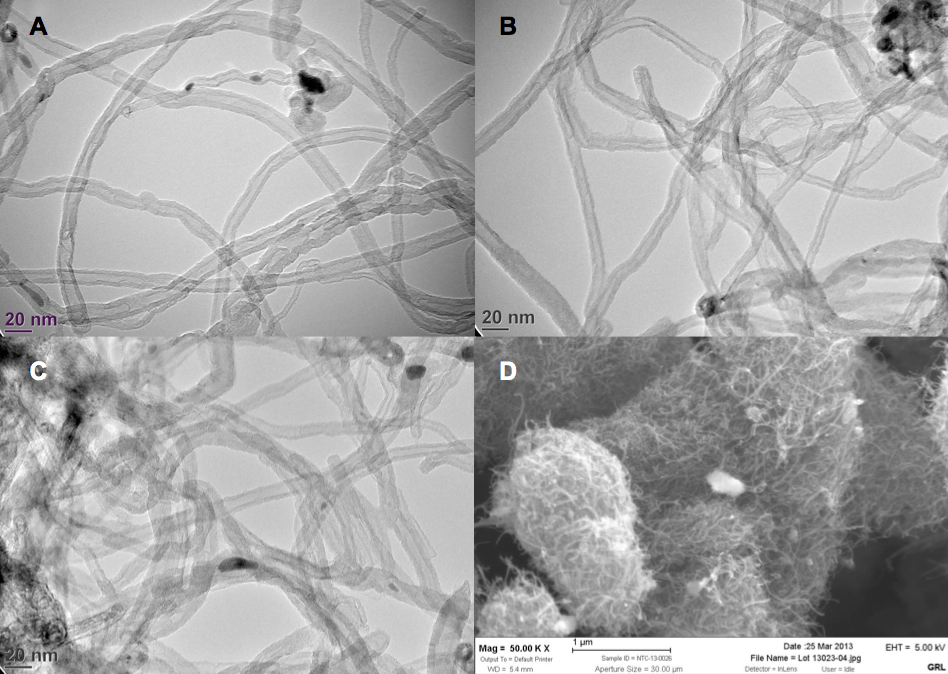


**Figure s5 - BALF parameters of male rats 24 hours (A) and 4 weeks (B) after a 5-day exposure to Graphistrength**^©^ **C100**

Changes are shown as x-fold differences compared to controls using a logarithmic scaling.

Abbreviations: ALP: alkaline phosphatase, GGT: γ-Glutamyltransferase, LDH: lactate dehydrogenase.

**
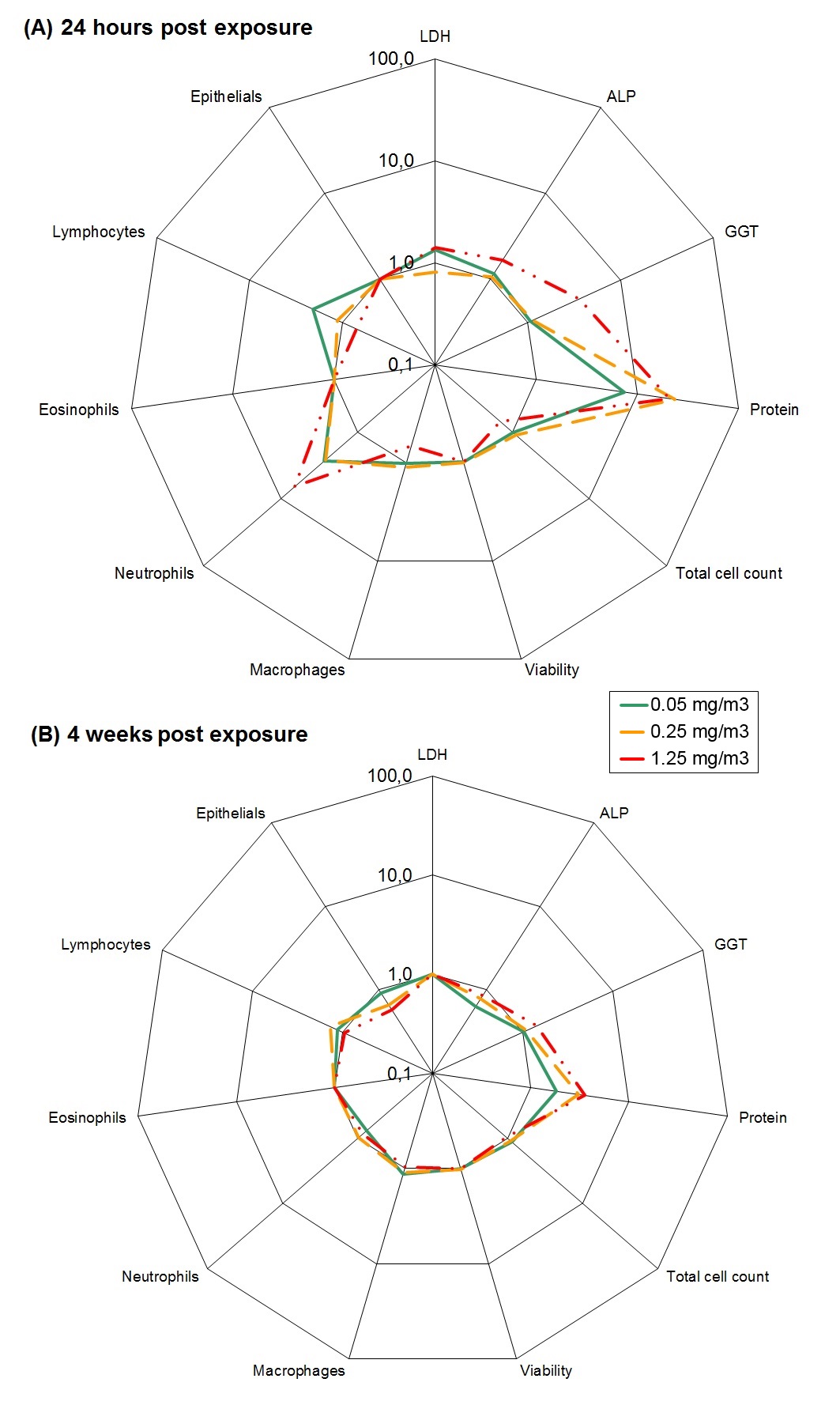
**

**Figure s6 –Microscopic appearance of lungs around terminal bronchiole in rats after a 5-day exposure to Graphistrength**^©^ **C100**

**(A)** Control. **(B)** Graphistrength^©^ C100, 1.30 mg/m^3^, after a 5-day inhalation exposure: Terminal bronchiolar epithelium showing hypertrophic appearance (asterisks). No inflammatory and granulomatous lesions are found. **(C)** Graphistrength^©^ C100, 1.30 mg/m^3^, after a 5-day inhalation exposure: Blackish particle laden alveolar macrophages (arrows). **(D)** Graphistrength^©^ C100, 1.30 mg/m^3^, after a 4-week recovery period: Histologic appearance of the bronchiolar epithelium recovered partially.

Original lens magnification: (A), (B) and (D) Magnification: 10 fold; (C) Magnification: 40 fold.


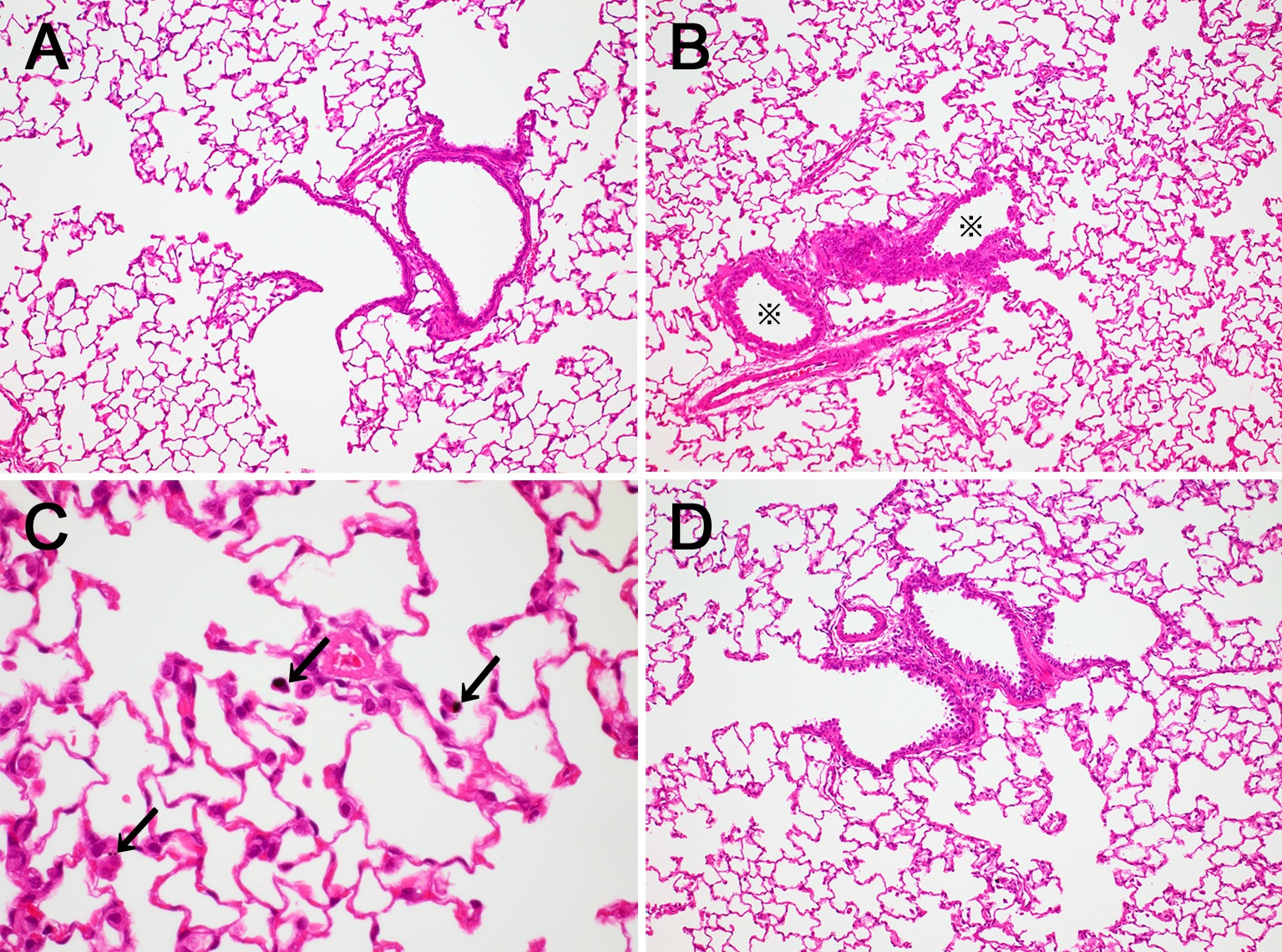


## TABLES

## Table s1 – Oxygen surface content by XPS in Graphistrength^©^ C100 aerosols from the rotating brush dust generator [16]

| **Origin of test material sample** | **Oxygen surface content (%)^1^** |
| --- | --- |
| As supplied (Batch no. 6097) | < 0.2^2, 3^ |
| Cyclonic separator | 1.0 ± 0.2 |
| Exposure chamber | 1.7 ± 0.3 |

^1^ mean ± sd of 3 repeated analysis

^2^ limit of detection

^3^ one analysis

## Table s2 – Effect by XPS of the milling duration and milling atmosphere (air or argon) on the oxygen surface content in Graphistrength^©^ C100

| **Duration of ball milling (h)** | **Oxygen surface content (%)^1^** | |
| --- | --- | --- |
|  | Standard Conditions (air) (Batch no. 8287) | Under Argon Atmosphere (Batch no. 110329-18) |
| 0 (test material as supplied) | 1.1 ± 0.2 | 0.5 ± 0.1 |
| 6 | 1.0 ± 0.1 | - |
| 24 | 1.2 ± 0.3 | - |
| 48 | 1.5 ± 0.1 | - |
| 96 | 2.1 ± 0.2 | 0.8 ± 0.3 |

^1^ mean ± sd of 4 repeated analysis

## Table s3 – Physico-chemical characterization of Graphistrength^©^ C100 before and after aerosol generation

|  | **Original Graphistrength**^©^ **C100** | | | **Graphistrength**^©^ **C100** milled 12 h under argon (63 µm sieving) | **Graphistrength**^©^ **C100** micronised and aerosolised collected at the exhaust of the elutriator |
| --- | --- | --- | --- | --- | --- |
| **Physico-chemical properties** | **Batch no. 6097** used in the published 5-day study [16] | **Batch no. 8287** used in this 5-day study | **Batch no. 110329-18** used in this 13-week study | | |
| Apparent Density (g/cm^3^) (mean ± sd) | 0.107^A^ | 0.085^A^ | 0.106 ± 0.06 (n = 3)^B^ | 0.2, 0.2^B^ | 0.17, 0.18^B^ |
| Elementary organic analysis^C^ |  |  |  |  |  |
| % C | nd^1^ | nd | 92.0, 91.6 | 91.1, 90.8 | 90.2, 90.1 |
| % H | nd | nd | < LoD^2^ | < LoD | < LoD |
| % N | nd | nd | < LoD | < LoD | < LoD |
| % O | nd | nd | < LoD | < LoD | < LoD |
| Ash content (%)^D^ | 8.6 | 8.6 | 8.2 ± 0.0 (n = 3) | nd | nd |
| Particle Size Distribution (µm)^E^ |  |  |  |  |  |
| D_10_ | 221 | 217 | 223 | nd | nd |
| D_50_ | 339 | 376 | 418 | nd | nd |
| D_90_ | 612 | 781 | 655 | nd | nd |
| Specific area (m²/g)^F^ | 141.1 | 187 ± 6 | 225.6 | 244 | 242 |
| Metal Content^G^ |  |  |  |  |  |
| Al (% w/w) | 3.4 | 3.2 | 3.0 ± 1.5 (n = 4) | 2.9, 3.0 | 3.0, 3.0 |
| Fe (% w/w) | 2.8 | 2.7 | 2.7 ± 0.6 (n = 4) | 2.2, 2.3 | 2.1, 2.1 |
| Co (ppm) | <10 | 18 | <50 | <10 | <10 |
| Cr (ppm) | 125 | 24 | <50 | 10 | 30 |
| Mn (ppm) | 15 | 12.5 | <50 | 10 | 15 |
| Mo (ppm) | <10 | <10 | <50 | <10 | <10 |
| Nb (ppm) | <10 | <10 | <50 | <10 | <10 |
| Ni (ppm) | 219 | 25 | <50 | 8 | 20 |
| Ti (ppm) | <10 | <10 | <50 | <10 | <10 |
| V (ppm) | <10 | <10 | <50 | <10 | <10 |
| W (ppm) | <10 | <10 | <50 | <10 | <10 |
| Chemical Surface Analysis by XPS^H^ |  |  |  |  |  |
| C (% w/w) | 99.2 ± 0.6 (n = 4) | 99.5 ± 0.3 (n = 4) | 99.5 ± 0.2 (n = 14) | 99.1 ± 0.2 (n = 4) | 99.2 ± 0.3 (n = 4) |
| O (% w/w) | 0.34 ± 0.05 (n = 4) | 0.43 ± 0.25 (n = 4) | 0.54 ± 0.20 (n = 14) | 0.70 ± 0.12 (n = 4) | 0.62 ± 0.22 (n = 4) |
| N (% w/w) | 0.17 ± 0.08 (n = 4) | 0.12 ± 0.08 (n = 4) | < 0.2 (n = 14) | < 0.2 (n = 4) | < 0.2 (n = 4) |
| Al (% w/w) | 0.16 ± 0.09 (n = 4) | 0.11 ± 0.12 (n = 4) | < 0.2 (n = 14) | 0.17 ± 0.06 (n = 4) | 0.13 ± 0.08 (n = 4) |
| Fe (% w/w) | 0.09 ± 0.04 (n = 4) | 0.06 ± 0.06 (n = 4) | < 0.2 (n = 14) | <0.1 (n = 4) | <0.1 (n = 4) |
| Diameters^I^ |  |  |  |  |  |
| External Diameters (nm) (mean ± sd) | 12.7 ± 4.2 | 11.8 ± 3.2 | 12.1 ± 3.5 | 12.1 ± 3.5 | 11.8 ± 3.0 |
| Internal Diameters (nm) (mean ± sd) | 5 ± 2.2 | 4.7 ± 1.7 | 4.4 ± 1.5 |  |  |
| Walls number (mean ± sd)^I^ | 12 ± 5 | 11 ± 3 | 12 ± 4 | 12 ± 5 | 12 ± 4 |
| Lenght (nm)^I^ |  |  |  |  |  |
| mean ± sd | 1007 ± 746 | 1050 ± 674 | 1069 ± 1102 | 713 ± 537 | 750 ± 623 |
| D_10_ | 330 | 310 | 209 | 201 | 182 |
| D_50_ | 820 | 910 | 708 | 569 | 563 |
| D_90_ | 2150 | 1950 | 2400 | 1434 | 1633 |
| Min. length | 168 | 50 | 90 | 96 | 86 |
| Max. length | 3780 | 3105 | 7200 | 2575 | 2926 |
| Surface to Volume ratio (m^-1^)^J^ | nd | nd | 2.4 ⋅10^7^ | 4.9 ⋅ 10^7^ | 4.2 ⋅ 10^7^ |
| Ends and alignment of Carbon Nanotubes (% open tips)^K^ | nd | nd | 20 | nd | 25 |

**A:** Porosimetry with mercury intrusion with an intrusion Autopore IV 9500 (Micromeritics France S.A., Verneuil en Halatte, France) with an adapted software (v1.05). **B:** Adapted from ISO 3923/2. Calculated by weighing the mass of 50 cm^3^ of MWCNT. **C:** The sample was introduced in an oven at a temperature of more than 1000°C under an oxygen flow. Combustion products were then driven by a helium flow on oxidization reagents and reduction reagents brought at high temperature. Carbon was then in the form of CO_2_, nitrogen as N_2_, and hydrogen in the form of H_2_O . After separation on a chromatographic column, these compounds were detected successively by a Katharometer. Hydrogen, nitrogen and oxygen were below the limit of detection. **D:** Adapted from ISO TR:10929/2012. Calcination at 800°C during 1 hour. Ratio between ash and product. **E:** Particle size distribution was measured with a Malvern Mastersizer S 2000 with a sample conditioner Qspec (MS17). After sieving of 10 g of MWCNT at 800 µm, 0.3 g of resulting powder was put in 30 mL of water and analysed with the following parameters: focale 300RF, calculation matrix 3OHD, beam width 2.4 nm and analysis model: polydisperse. **F:** Specific surface area was measured using an ASAP 2000 from MICROMERITICS Company with two degassing stations in compliance with NF ISO 9277 standard. **G:** Adapted from ISO/TS 13278:2011. Measured with a ICP/AES OPTIMA 4300 DV PERKIN ELMER. Digestion of the sample was made by lithium tetraborate fusion method. **H:** XPS analysis was made with a photoelectron spectrometer SSX 100 (Surface Science Laboratories - VG Instruments- Fisons Instruments). Size of the analysed area was of about 400 x 800 µm². A survey spectrum (from 10 eV to 1200 eV) was made in a first time and a detailed spectrum afterwards targeted on energies corresponding to the detected elements. **I:** Adapted from ISO/TF 10929:2012 and ISO/IEC PDTS 10797:2011. Philips - FEI CM 200 microscope was used with a LAB6 filament - maximum voltage 200 kv, point to point resolution 0.27 nm. Data were calcultated from the measurement of at least 100 MWCNT. **J:** Obtained by multiplying the specific surface area by apparent density. **K:** TEM achieved at different magnifications was used in order to evaluate the quality of MWCNT, the tube ends and the alignment along one axis of carbon nanotubes.

^1^ not determined

^2^ LoD: limit of detection. 0.2% for H, 0.4% for N and 0.3% for O

**Table s4 - Target and achieved aerosol concentrations and particles size of Graphistrength**^©^ **C100. Temperature, relative humidity and oxygen concentration measured over the 5-day exposure study**

| **Groups** | **Control** | **Low** | **Mid** | **High** |
| --- | --- | --- | --- | --- |
| Target aerosol concentration (mg/m^3^ air) | 0 | 0.05 | 0.25 | 1.25 |
| Achieved aerosol concentration (mg/m^3^ air) | - | 0.066 ± 0.036 (n=5) | 0.26 ± 0.03 (n=5) | 1.30 ± 0.03 (n=5) |
| Particle size MMAD (µm) (gravimetric determination) ^1^ | - | 1.93 / 2.56 (n=2) | 1.97 (n=1) | 1.84 / 1.85 (n=2) |
| Range of GSD (gravimetric determination)^1^ | - | 1.30 / 4.33 (n=2) | 1.94 (n=1) | 1.76 / 1.93 (n=2) |
| Mean percentage of particles < 3 µm (gravimetric determination) | - | 68.4^1^ | 73.6 | 78.3 |
|  | | | | |
| Mean temperature (°C) | 23.3 ± 0.0 | 22.9 ± 0.0 | 23.0 ± 0.1 | 23.0 ± 0.0 |
| Mean relative humidity (%) | 7.4 ± 0.3 | 0.1 ± 0.2 | 0.9 ± 0.2 | 4.9 ± 0.2 |
| Mean oxygen concentration (%) | 20.6 ± 0.0 | 20.6 ± 0.0 | 20.5 ± 0.1 | 20.6 ± 0.0 |

^1^ aerosols for particle sizes determination were sampled at a high air flow rate of 9 L/min at a representative animal exposure port.

## Table s5 - Cellular analysis of BALF of male rats 24 hours and 4 weeks after a 5-day exposure to Graphistrength^©^ C100

| **Concentration** (mg/m^3^) | **0** | **0.05** | **0.25** | **1.25** |
| --- | --- | --- | --- | --- |
| **24 hours post exposure** | | | | |
| Total cell count (10^6^) | 4.92 ± 1.46 | 5.04 ± 0.68 | 5.53 ± 0.65 | 3.45 ± 0.89 |
| Viability (%) | 97.1 ± 0.5 | 95.2 ± 2.7 | 96.2 ± 0.8 | 95.1 ± 1.2 |
| Macrophages (%) | 98.3 ± 0.5 | 97.0 ± 1.6 | 97.3 ± 2.2 | 95.0 ± 3.3 |
| Neutrophils (%) | 0.3 ± 0.3 | 0.8 ± 0.9 | 0.7 ± 1.2 | 2.8 ± 2.7* |
| Eosinophils (%) | 0.0 ± 0.1 | 0.1 ± 0.2 | 0.1 ± 0.2 | 0.1 ± 0.1 |
| Lymphocytes (%) | 0.4 ± 0.2 | 0.8 ± 0.5 | 0.4 ± 0.5 | 0.4 ± 0.5 |
| Epithelials cells (%) | 0.6 ± 0.3 | 1.0 ± 0.2 | 1.2 ± 0.5 | 1.2 ± 0.8 |
| Others (%) | 0.4 ± 0.1 | 0.4 ± 0.1 | 0.3 ± 0.2 | 0.4 ± 0.2 |
| Macrophages with phagocyted material (%) | - | 1.8 ± 0.8 | 8.5 ± 2.3 | ca. 50 |
| **4 weeks post exposure** | | | | |
| Total cell count (10^6^) | 5.75 ± 0.97 | 6.58 ± 1.29 | 6.33 ± 0.97 | 5.50 ± 0.67 |
| Viability (%) | 96.0 ± 3.4 | 96.3 ± 1.5 | 97.2 ± 0.8 | 96.8 ± 1.4 |
| Macrophages (%) | 96.8 ± 1.3 | 97.6 ± 1.4 | 97.4 ± 1.6 | 97.7 ± 1.4 |
| Neutrophils (%) | 0.9 ± 0.9 | 0.6 ± 0.8 | 0.8 ± 0.7 | 0.8 ± 0.7 |
| Eosinophils (%) | 0.0 ± 0.1 | 0.0 ± 0.0 | 0.1 ± 0.1 | 0.0 ± 0.0 |
| Lymphocytes (%) | 0.4 ± 0.5 | 0.4 ± 0.3 | 0.5 ± 0.4 | 0.4 ± 0.3 |
| Epithelials cells (%) | 1.0 ± 0.3 | 0.8 ± 0.3 | 0.6 ± 0.3 | 0.6 ± 0.5 |
| Others (%) | 0.8 ± 0.7 | 0.6 ± 0.5 | 0.6 ± 0.6 | 0.5 ± 0.3 |
| Macrophages with phagocyted material (%) | - | 1.1 ± 0.3 | 4.9 ± 1.0^#^ | 16.5 ± 8.1^##^ |

* p<0.05; ** p<0.01; statistically significant differences to controls.

# p<0.05; ## p<0.01; statistical significance by comparison to the 24-hour post exposure at the same concentration.

## Table s6 - Biochemical analysis of BALF of male rats 24 hours and 4 weeks after a 5-day exposure to Graphistrength^©^ C100

| **Concentration** (mg/m^3^) | **0** | **0.05** | **0.25** | **1.25** |
| --- | --- | --- | --- | --- |
| **24 hours post exposure** | | | | |
| LDH (U/L) | 6.0 ± 3.4 | 8.0 ± 1.4 | 4.9 ± 4.6 | 8.4 ± 1.5 |
| ALP (U/L) | 32.1 ± 7.8 | 37.0 ± 20.2 | 33.9 ± 12.7 | 53.5 ± 12.5 |
| GGT (U/L) | 3.2 ± 1.0 | 3.4 ± 1.7 | 3.6 ± 1.1 | 11.4 ± 3.2* |
| Protein (mg/L) | 40 ± 50 | 300 ± 80 | 950 ± 400** | 830 ± 110** |
| **4 weeks post exposure** | | | | |
| LDH (U/L) | 1.6 ± 3.2 | 0.0 ± 0.0 | 0.0 ± 0.0 | 0.0 ± 0.0 |
| ALP (U/L) | 27.3 ± 4.8 | 17.3 ± 4.1 | 21.3 ± 10.3 | 23.6 ± 4.6 |
| GGT (U/L) | 4.0 ± 1.1 | 4.1 ± 0.8 | 4.4 ± 0.8 | 5.7 ± 0.8 |
| Protein (mg/L) | 210 ± 110 | 380 ± 130 | 640 ± 310* | 750 ± 130** |

* p<0.05, ** p<0.01; statistically significant differences to controls.

**Table s7 – Main microscopic findings in the lungs of male and female rats 24 hours and 4 weeks after a 5-day exposure to Graphistrength**^©^ **C100**

|  | **MALES** | | | |  | **FEMALES** | | | |
| --- | --- | --- | --- | --- | --- | --- | --- | --- | --- |
| **Concentration** (mg/m^3^) | **0** | **0.05** | **0.25** | **1.25** |  | **0** | **0.05** | **0.25** | **1.25** |
| Number examined  (24-hour/4-week post exposure) | 5/5 | 5/5 | 5/5 | 5/5 |  | 5/5 | 5/5 | 5/5 | 5/5 |
| *Alveolar infiltration of macrophages* |  |  |  |  |  |  |  |  |  |
| minimal | 5/5 | 5/5 | 5/5 | 2/1 |  | 4/4 | 5/5 | 5/5 | 2/1 |
| slight | -/- | -/- | -/- | 3/4 |  | -/1 | -/- | -/- | 3/4 |
| **Mean severity^1^** | **1.0/1.0** | **1.0/1.0** | **1.0/1.0** | **1.6/1.8** |  | **0.8/1.2** | **1.0/1.0** | **1.0/1.0** | **1.6/1.8** |
| *Black inclusion in macrophage cytoplasm* |  |  |  |  |  |  |  |  |  |
| minimal | -/- | 5/5 | 1/3 | -/ |  | -/- | 5/5 | 1/1 | -/- |
| slight | -/- | -/- | 4/2 | -/ |  | -/- | -/- | 4/4 | -/- |
| moderate | -/- | -/- | -/- | 5/5 |  | -/- | -/- | -/- | 5/5 |
| **Mean severity^1^** | **-/-** | **1.0/1.0** | **1.8/1.4** | **3.0/3.0** |  | **-/-** | **1.0/1.0** | **1.8/1.8** | **3.0/3.0** |
| *Bronchial/bronchiolar epithelial cells hypertrophy* |  |  |  |  |  |  |  |  |  |
| minimal | -/- | -/- | -/- | 3/2 |  | -/- | -/- | -/- | 1/2 |
| slight | -/- | -/- | -/- | 1/- |  | -/- | -/- | -/- | 1/- |
| **Mean severity^1^** | **-/-** | **-/-** | **-/-** | **1.0/0.4** |  | **-/-** | **-/-** | **-/-** | **0.6/0.4** |

Histopathological findings were graded in severity using a five point system of minimal (grade 1), slight (grade 2), moderate (grade 3), marked (grade 4) or severe (grade 5)

^1^: mean severity is ∑ number of animals x severity / number of examined organs in the group

- : no animal affected
